# Limitation of seedling growth by potassium and magnesium supply for two ectomycorrhizal tree species of a Central African rain forest and its implication for their recruitment

**DOI:** 10.1002/ece3.1835

**Published:** 2015-12-15

**Authors:** Godlove Ambe Neba, David McClintock Newbery, George Bindeh Chuyong

**Affiliations:** ^1^ Department of Botany and Plant PhysiologyUniversity of BueaP. O. Box 63BueaS. W. RegionCameroon; ^2^ Institute of Plant SciencesUniversity of BernAltenbergrain 21CH‐3013BernSwitzerland

**Keywords:** Growth, magnesium: potassium, recruitment limitation, tree seedlings, tropical forest

## Abstract

In the ectomycorrhizal caesalpiniaceous groves of southern Korup National Park, the dominant tree species, *Microberlinia bisulcata*, displays very poor in situ recruitment compared with its codominant, *Tetraberlinia bifoliolata*. The reported ex situ experiment tested whether availabilities of soil potassium and magnesium play a role. Seedlings of the two species received applications of K and Mg fertilizer in potted native soil in a local shade house, and their responses in terms of growth and nutrient concentrations were recorded over 2 years. Amended soil concentrations were also determined. *Microberlinia* responded strongly and positively in its growth to Mg, but less to K; *Tetraberlinia* responded weakly to both. Added Mg led to strongly increased Mg concentration for *Microberlinia* while added K changed that concentration only slightly; *Tetraberlinia* strongly increased its concentration of K with added K, but only somewhat its Mg concentration with added Mg. Additions of Mg and K had small but important antagonistic effects. *Microberlinia* is Mg‐demanding and apparently Mg‐limited in Korup soil; *Tetraberlinia*, whilst K‐demanding, appeared not to be K‐limited (for growth). Added K enhanced plant P concentrations of both species. Extra applied Mg may also be alleviating soil aluminum toxicity, and hence improving growth indirectly and especially to the benefit of *Microberlinia*. Mg appears to be essential for *Microberlinia* seedling growth and its low soil availability in grove soils at Korup may be an important contributing factor to its poor recruitment. *Microberlinia* is highly shade‐intolerant and strongly light‐responding, whilst *Tetraberlinia* is more shade‐tolerant and moderately light‐responding, which affords an interesting contrast with respect to their differing responses to Mg supply. The study revealed novel aspects of functional traits and likely niche‐partitioning among ectomycorrhizal caesalps in African rain forests. Identifying the direct and interacting indirect effects of essential elements on tropical tree seedling growth presents a considerable challenge due the complex nexus of causes involved.

## Introduction

The Atlantic Coastal Forest of Central Africa is a spatially and temporally complex forest type characterized by patches of varying extent in which species of the subfamily Caesalpiniaceae (family Fabaceae = Leguminosae) dominate compositional abundance (Letouzey 1968, 1985). A great majority of these species are in the tribe Amherstieae and accordingly they are ectomycorrhizal (EM) (Newbery et al. 1988). A question central to understanding the ecology of this African forest is how, locally and regionally, these relatively few EM species have come to be much more abundant than the many non‐EM ones (Gartlan et al. 1986; Newbery and Gartlan 1996; Newbery et al. 1997). At Korup in southwest Cameroon, three caesalps species, *Microberlinia bisulcata* A. Chev., *Tetraberlinia bifoliolata* Harms (Haumann), *and Tetraberlinia korupensis* Wieringa, codominate in groves (large patches), among which *M. bisulcata* is the most abundant in terms of basal area and defines grove perimeter (Newbery et al. 2004, 2013). Korup lies within the Guinea–Congolean refugium, one of the last large and intact remnants of the Atlantic Coastal Forest (Gartlan 1992). This lowland tropical rain forest stands on strongly weathered, well‐draining, and nutrient‐poor, sandy soils at an elevation of 50–150 m above sea level.

Despite the apparent high degree of adaptation of adult *M. bisulcata* trees to the site, and copious seed and seedling production every 2–3 years on mast fruiting (Green and Newbery 2002; Newbery et al. 2006a, 2009; Norghauer and Newbery 2015), *M. bisulcata* is nevertheless not replacing itself either within or outside the groves (Newbery et al. 1997, 2004). This is evidenced by the high seedling mortality, and resulting in very low densities of sapling and juvenile trees (Newbery et al. 2006b, 2010, 2013). By contrast, *T. bifoliolata* and *T. korupensis* do show good regeneration in terms of seedling, sapling, and juvenile tree densities. This situation has naturally been the subject of intensive research since the mid‐1980s, leading to a new hypothesis of ‘transient dominance’ explaining the current frequency distribution of tree sizes of *M. bisulcata* (Newbery et al. 2013). Norghauer and Newbery (2010) showed that establishment limitation was 2–4‐fold stronger than seed limitation in *M. bisulcata*, whereas seed limitation was always stronger than seedling limitation in the codominant *T. bifoliolata*.

Seedlings of *M. bisulcata* are highly shade‐intolerant and highly light‐responsive (Green and Newbery 2001a,b), and saplings can survive well in the open nursery conditions (D. M. Newbery, unpubl.). By contrast, the two *Tetraberlinia* species are much more shade‐tolerant and conversely respond less fast to increasing light levels. The relatively small seeds of *M. bisulcata* (at 0.64 g, 40–50% the dry mass of those of the *Tetraberlinia* spp.; Green and Newbery 2001a), and their postdispersal susceptibility to fungal infection and animal predation largely in the understorey (Green and Newbery 2002; Norghauer and Newbery 2011), although leaf herbivore attack on seedlings occurs mainly under lighted gap conditions (Green and Newbery 2001b; Norghauer and Newbery 2013, 2014), all play important roles in seedling survival. Neither leaf pathogens (Norghauer et al. 2010) nor soil phosphorus limitation (Newbery et al. 2002) affected seedling growth and survival. And whilst EM infection occurred on seedlings both close to and away from adults (no effect of adult basal area abundance), being infected had little effect on survival in their first 2 years (Newbery et al. 2000). Nitrogen, judging from seedling leaf concentrations, gave no indication of limiting growth and survival in *M. bisulcata* either (Newbery et al. 2000, 2002; Green and Newbery 2001a,b); levels in soil and litter, likely because of generally high N‐fixing activity in the forest, also did not suggest N‐limitation (Newbery et al. 1997; Chuyong et al. 2002). Of the untested macro‐elements, however, apart from calcium, and ones that might be essential for growth response to light, were potassium and magnesium.

Across the groves and into the surrounding forest matrix of mixed tree species, the deep and, in places stony, sandy acidic quartzite soils have atop them a thin well‐marked 1‐ to 2‐cm layer of organic matter, fine roots, and EM hyphae (Newbery et al. 1988, 1997, 2000;). The intense May–October wet season at Korup (annual rainfall *c*. 5.5 m, reaching 0.8–1.0 m in August alone), set between the equally marked single December–March dry season (Newbery et al. 1998, 2002, 2006a), means not only a considerable leaching of the canopy and of the soils, but also that this throughfall is coupled with lagged effects of the annual dry‐season peak in litterfall (Chuyong et al. 2000, 2002, 2004). The organic layer is clearly essential for the efficient recycling of N and P through decomposition (Newbery et al. 1997) and it is surmised – and currently under test – that the layer is essential too for the trapping and reuptake of potassium (K) and magnesium (Mg) from this throughfall (G. A. Neba, thesis in prep. and unpubl. data). Litter‐bag trials at Korup (Chuyong et al. 2002) indicated that among the five macronutrients, just Mg showed significantly much higher mineralization rates in grove than nongrove soils, indicative of a large demand by predominantly adult *M. bisulcata* trees for Mg, and in turn that available levels of Mg would be reduced for seedlings around adults. This new insight led to the hypothesis that Mg could be a potentially limiting factor for *M. bisulcata* regeneration. The laterally extensive buttressing on *M. bisulcata* is thought to allow large trees nutrient exploration of very large areas of surface soil in the forest (Newbery et al. 2009).

Gartlan et al. (1986) reported a close association between low phosphorus (P) and low K concentrations in soils of the grove‐forming EM species in Korup. Whilst P has received much attention because of the putative role of EM species in enhancing its availability to, and acquisition by, trees in low‐P soils (Newbery et al. 1997, 2002), the potential role of K in the Korup ecosystem and as a controlling factor of seedling recruitment has been overlooked. Its nutrient transfer, operating to a large degree also via throughfall, gives its nutrient cycling strong temporal parallels with Mg, although the quantities involved are eightfold higher for K than Mg (Chuyong et al. 2004). Furthermore, how availability of K affects seedlings and their EM development, and thereby the efficacy in P uptake, is unknown; it is also unclear how K and Mg may interact in their possible limitations of seedling growth. Thus, a comparison of responses by the light‐responsive *M. bisulcata* and one of the shade‐tolerant codominant *Tetraberlinia* spp. to K and Mg supply at levels relevant to their ecology and conditions in the forest was prompted.

The specific aim was to evaluate the effects of Mg and K in various combinations of application level on seedling growth, allocation of dry matter, and nutrient status. The more general aim, however, was to use the results to reach a deeper understanding of whether the two species might be partitioning the forest niche with regard to their use of the elements in early growth. If Mg and K are varying spatially across the forest, as recent findings suggest (G. Neba, unpubl. data), this process might lead to one species recruiting better than the other at different locations (Newbery et al. 2006b, 2010). The experiment set out, therefore, to test a new hypothesis concerning regeneration in the groves of codominant caesalpiniaceous tree species at Korup.

The shade‐house experiment reported here used *T. bifoliolata* as the second species because its seeds were available along with those of *M. bisulcata* in the start year. *T. korupensis* would have been a good alternative had it seeded (Newbery et al. 1988, 2000, 2013). The two selected species will be referred to from here on by just their generic names.

## Methods

### Experimental design and setup

The experiment was conducted at an open nursery site within the PAMOL plantation near the footbridge over the Mana River, at the entrance to Korup National Park. This 0.3‐ha research site of the University of Bern was set up in 1995 by the clearance of the then 4‐year‐old palms. It had previously supported mature palms, and had received rock phosphate fertilization up until 1982 (Green and Newbery 2001a,b). The substrate, soil, and climatic parameters for the site are otherwise very similar to those of the southern part of the Park, where all the ecological field work has been conducted. Soil of the nursery site was not used in the experiment itself and only as a bedding medium.

Two seed beds were prepared inside of a mesh‐covered shade house (Tildenet, UK) that allowed 25% transmission of external radiation. The bedding soil was inoculated with 10 kg of fresh organic matter collected from below the litter in the top 1–2 cm of the soil profile from under the canopies of six large *Microberlinia* trees in the 82.5‐ha permanent P‐plot, situated within the main grove at Korup (Newbery et al. 2004, 2013), to ensure suitable ectomycorrhizal inoculation of seedlings. Over 600 freshly collected (fungus‐free) seeds of each species were sown on 5 August 2010. They came from under 30 fruiting *Microberlinia* and six *Tetraberlinia* trees across the main Korup grove. Nursery bags were filled with well‐mixed top soil (5.0 kg oven dry weight per bag) excavated from under *Microberlinia* trees in a 6.75‐ha experimental plot inside another smaller grove lying just outside the southern boundary of the Park, at Isangele Road (Newbery et al. 2002). The surface litter was carefully moved aside and soil taken to a depth of 5–7 cm, which included the thin surface organic layer with larger roots removed. Eight hundred 10‐week‐old seedlings from the nursery bed were individually transplanted into standard 20‐cm diameter and 25‐cm deep black polyethylene nursery bags on 10 November 2010 and allowed to establish. Seedlings were irrigated regularly during the dry season with water from the Mana River.

The experiment was laid out in a randomized block design. On 6 January 2011, each seedling in its bag was placed on a plastic plate and these arranged on benches raised 0.7 m above ground in the shade house (Fig. S1). Between 5 and 12 April 2011, surviving seedlings, now 7.5 months old, were sorted according to size and arranged into four blocks, with block 1 having the largest seedlings and block 4 the smallest ones. The basic experimental unit for the treatments consisted of 16 seedlings, replicated four times to give a total of 64 seedlings per species, and 128 seedlings for the two species in each of the four blocks. Initial size was recorded in terms of seedling height and leaf and branch numbers before applying the treatments.

The treatments consisted of two factors, potassium (K) and magnesium (Mg), each at four increasing levels (L1 to L4) in a crossed structure. Potash as the source of K was obtained from the local market, while kieserite as the source of Mg was obtained from a commercial company (Yara Cameroon), and both put through quality control testing at the Universities of Dschang and Bern. Impurity loads in the samples were negligible. The amounts to be applied were calculated from the concentrations of the fertilizers as follows. The proportion of K_2_O in potash was 50.11%, and accordingly the levels of K applied were: (1) no fertilizer; (2) 5 g potash > 2.08 g K (low); (3) 10 g potash > 4.16 g K (intermediate); and (4) 15 g potash > = 6.24 g K (high). The proportion of MgO in kieserite was 27.65%, and likewise the levels of Mg applied were: (1) no fertilizer; (2) 5 g kieserite > 0.83 g Mg (low); (3) 10 g kieserite > 1.68 g Mg (intermediate); and (4) 15 g kieserite > 2.49 g Mg (high). The larger range in amounts of K compared with Mg applied was done to reflect the relatively 2‐ to 3‐fold higher available concentrations of K to Mg in organic matter/top mineral soil at the P‐plot and Isangele Road (see later Results). The seedlings were numbered from 1 to 256 for each species and the treatments allocated at random within blocks. Fertilizers were spread dry on the surface of each pot, making sure that they did not touch the stems of seedlings, and watered in well. Two equal rounds of fertilizer application were made in exactly the same manner, one on 12 April 2011, and the other on 18 January 2012.

Estimates of solar radiation were available from the PAMOL meteorological station at Bulu, 4 km from Mana Bridge. Due to a failure of the Gunn–Bellani radiometer, relevant data were available only from 30 May 2011 to the end of the experiment. Estimates for 4 April 2011 (day prior to start of experiment) and up to this date in May were achieved by substituting averages of the matching daily values of 2006–08.

### Plant and soil measurements

From 20 April 2011, seedlings were measured at approximately monthly intervals, at which times pots were randomly relocated within blocks: on the 19 occasions (the 9th, 14th, and last were just before the harvests), plant height, leaf number, and branch number were recorded for each survivor. Later at harvests 2 and 3, basal stem diameter (just above ground, point paint‐marked) and leaf area were measured. The three harvests were on 10–17 January 2012 (H1), 3–10 July 2012 (H2), and 2–14 December 2012 (H3), each marking the end of a growth phase. Some seedlings suffered a shock in terms of sudden temporary leaf shedding 1 month after the first fertilizer application.

At each harvest, 64 seedlings per species were randomly selected such that one replicate seedling per treatment was taken from each block. Leaves were removed and placed in paper bags. A soil sample was taken from the upper and lower layers of each pot at harvests 1 and 2. Soil samples were later air‐dried and sieved (2‐mm mesh) for chemical analysis at Bern. Each pot was then left in water for about 10 min and the soil carefully washed from the roots of seedlings. Roots of *Microberlinia* were visually scored for ectomycorrhizal abundance by estimating the proportion of root tips colonized; for *Tetraberlinia* this was not possible because, unlike on *Microberlinia*, the fungal sheaths are much less obvious without a microscope. Washed seedlings were separated into roots and stems, and bagged.

Plant samples from the first harvest were oven‐dried in Mundemba (65°C for 72 h) before transport to the University of Buea, redried, and weighed (Ohaus, electronic balance; ±0.01 g). Second‐ and third‐harvest samples were treated in similar manner, except that they were transported fresh to Buea and their leaf areas measured (Delta‐T, Cambridge, UK) before drying. All leaf samples were later milled, as were roots and stems from just the second harvest, and in Bern, 300 mg each was digested in a 2.5‐mL mixture of selenium, sulphuric acid, and salicylic acid, and analyzed for total Ca, K, and Mg using the Inductively Coupled Plasma Optical Emission Spectrometer (ICP – OES; model Optima 7000DV), and total N and P on a Continuous Flow Optical‐Absorption Spectrometer (CF‐OAP; Skalar Scan^+^). Soil samples were also oven‐dried (65°C overnight), extracted in 1M ammonium acetate, filtered, and analyzed for the three cations in the same way as the plant materials.

### Statistical analysis

A principal component analysis (PCA) of initial height and leaf number provided a starting size covariate. Counts of leaf number (*lvn*_t_) in growth phase 1 were used to form a measure of leaf loss, or ‘seedling shock’. This shock covariate was defined as:

$$\text{shock}=\frac{\text{mean}\left(lvn_{1},lvn_{3},lvn_{4}\right)-lvn_{2}}{\text{mean}(lvn_{1},lvn_{3},lvn_{4})}$$

where *lvn*_*x*_ is leaf number at time x. Strongly, or severely, affected seedlings were those for which shock was, respectively, ≥ 0.5 yet <1.0, or equal to 1.0. The 4‐month period integrated any shock intensification and/or recovery.

Growth measurements (height, leaf number, stem diameter) within the three phases did not require transformation and were subject to repeated measures analysis of variance (REM‐ANOVA), for each species separately, using GenStat version 16 (Payne et al. 2011). Factorial ANOVA (species separately again) was used to analyze harvest dry mass (*ln*‐transformed) with and without starting size and shock covariates individually. Just one plant of *Microberlinia* died and was irreplaceable by harvest 3; any lost replicates could normally be substituted from the extra quarter of plants that remained not harvested by the end of the experiment. As the first covariate had scarcely any impact on the results for either species, but the second one often did, so this shock one was retained for all ANOVAs. Factorial ANOVA was also applied to leaf nutrient concentrations (*ln*‐transformed), but only for stem and root nutrients from harvest 2. There were no obviously outlying values for dry weights or plant nutrient concentrations. For the soil nutrient concentrations (again *ln*‐transformed), data for upper and lower layers were analyzed separately because they were not statistically independent. Here there were a few very high concentrations, likely due to contamination, and these were treated as missing (four K and Ca in *Microberlinia,* and four K in *Tetraberlinia,* pots; >4‐fold their means). Least significant differences between means (*P *=* *0.05) enabled treatment comparisons.

## Results

### Growth responses

**Figure 1:**
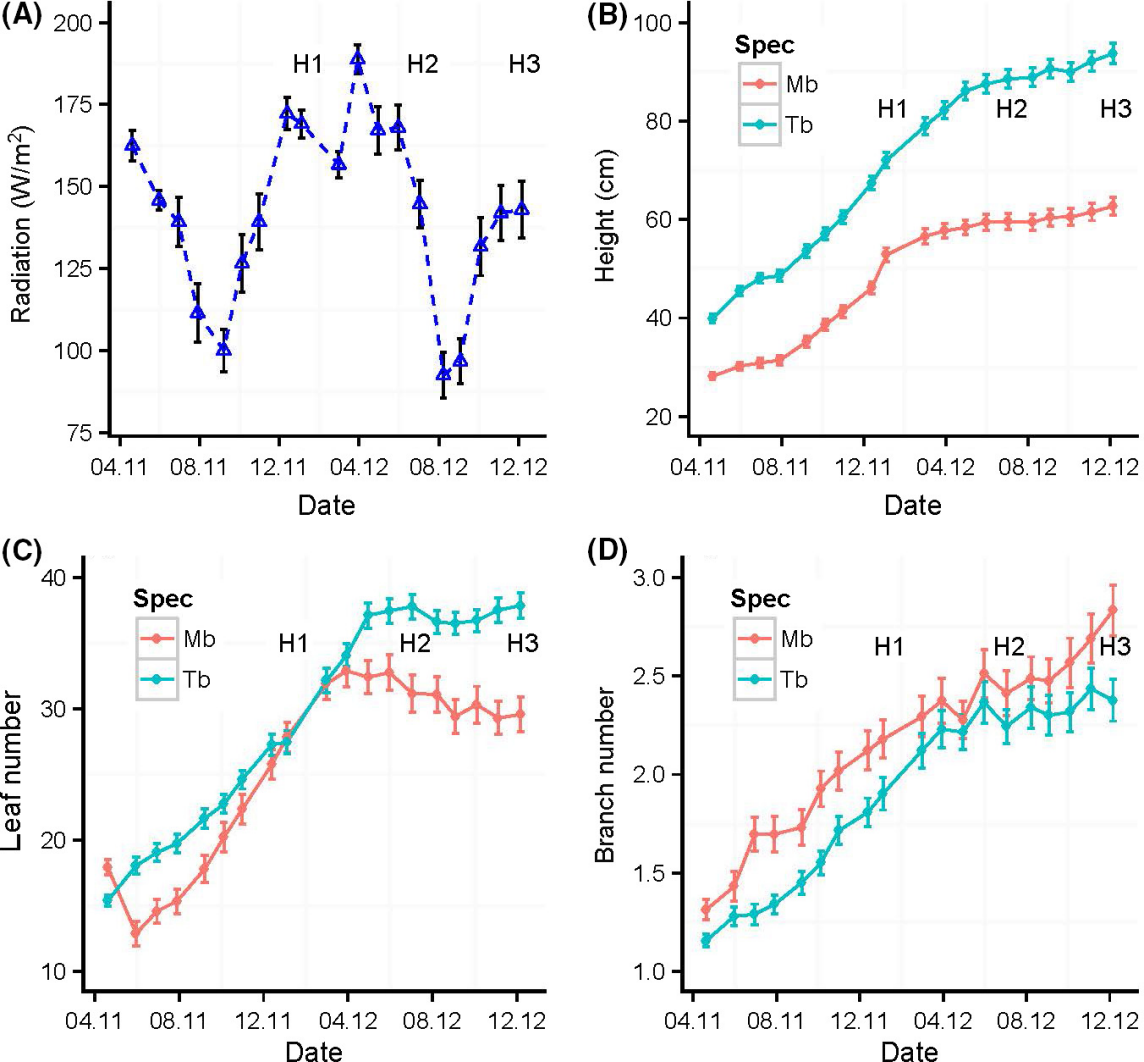
Variation in radiation during the course of the experiment (A) and the mean growth of *Microberlinia bisulcata* (Mb) and *Tetraberlinia bifoliolata* (Tb) in terms of (B) Height, (C) Leaf number, and (D) Branch number, across all treatments and for those plants that were present from start to finish. The labels H1, H2, and H3 indicate the timing of the harvests. Half of these plants (one of the four replicates) contributed to H3 at the end, whilst H1 and H2 used the two other replicates not part of the data presented here. Bars on points are ±1SE.

Over the course of the experiment, radiation changed according to the normal seasonal cycle at Korup (Fig. 1A), so that the start and end of seedling growth would have followed the same annual pattern that wildings in the forest would have experienced, albeit in the latter case at lower levels on average than in the nursery (Green and Newbery 2001b). Period 1 therefore spanned a first wet season with its typical steep drop in radiation in August–September, period 2 a dry season with a peak in December, and period 3 again a wet season (Fig. 1A). Averaging daily values for the individual time intervals corresponding to the 19 nondestructive measurements, means (±SD) of radiation for periods 1, 2, and 3 were 140.7 ± 25.0 (*n *=* *9), 165.1 ± 16.3 (*n *= 5), and 121.2 ± 24.7 (*n *=* *5), respectively. Thus, between harvests 2 and 3, the seedlings experienced a substantial 26.6% fall in radiation.

Whilst height and branch number continued to rise steadily until the time of harvest 3 (Fig. 1B and D), leaf number for *Microberlinia* decreased after harvest 1 (in periods 2 and 3), and for *Tetraberlinia* it became constant after midway between harvests 1 and 2 (within period 2, Fig. 1C). These trends are based on the 50% of plants (i.e., two out of the four replicates) that were left to grow to the end of the experiment. *Tetraberlinia* attained greater height and had more leaves than *Microberlinia*, although it branched less (Fig. 1B–D). The shock effect is well evident at the second date from the temporary drop in leaf numbers on *Microberlinia* (Fig. 1C): had this not occurred, leaf numbers for both species would probably have been very similar up to harvest 1. Even so, the later decline in leaf number for *Microberlinia*, as well as its lessening of height growth and branching rates, actually began when radiation was at its highest (Fig. 1A). Using independently sampled seedlings, dry mass continued to increase significantly, although leaf number and area decreased between harvests 2 and 3, in contrast to *Tetraberlinia*, which later increased leaf area despite little change in its leaf number. Mean ectomycorrhizal score across the 16 treatments was 23% (range 13–38%), significantly higher in the control than for K addition (*P *<* *0.01), but unaffected by Mg application and harvest (*P *>* *0.05).

Of the 256 *Microberlinia* seedlings, 30 (11.7%) suffered strong shock, 27 (10.5%) were severely shocked, and four (1.6%) died as a consequence. By comparison, only six (2.3%) *Tetraberlinia* seedlings suffered strong shock, of which one (0.4%) was severely shocked and three (1.2%) died. Of these shocked *Microberlinia* and *Tetraberlinia* seedlings, 92 and 90%, respectively, had received K‐addition levels 3 and 4. Shock values for *Microberlinia* increased significantly with increasing K level (*P *<* *0.001), but showed only a weak decrease with Mg level (*P *=* *0.36). For *Tetraberlinia*, no significant trend was apparent for either factor.

*Microberlinia* seedlings treated with fertilizer (L2–L4) performed better than control (L1) seedlings in all growth parameters and during all growth phases. Repeated measures ANOVA showed that seedlings of this species with Mg application had highly significant increases in height, leaf number, and stem diameter. K application, however, produced only marginally significant increases (Table S1). *Tetraberlinia* seedlings with Mg application showed improved growth compared with controls, but significantly so only for diameter, whilst K application resulted in growth reductions, again only significantly for diameter (Table S1). Interactions between K and Mg rarely produced significant differences for growth of either species. Average leaf area per leaf for *Microberlinia* was only marginally higher at Mg level 3 than the control (*P *<* *0.05) and showed no differences regarding K addition; *Tetraberlinia* showed no effects of Mg and K as factors (*P *>* *0.05).

### Dry mass responses

For *Microberlinia,* seedlings with Mg addition produced highly significantly more dry matter (DM) in total and per plant part than the controls (L1): the differences were most noticeable for the first application level (L2) vs. the control (L1), compared with the relatively smaller increases caused by higher applications (L2 to L3/L4). With K‐treated seedlings, only the stem (DMS) and total dry masses (DMT) were marginally significantly higher than control seedlings (Table 1). For *Tetraberlinia*, Mg application resulted in relatively small effects on dry mass, with weakly significant increases in roots (DMR) and total dry mass only. By contrast, *Tetraberlinia* seedlings with K application showed barely any effect on leaf dry mass and decreases in stem, root, and total dry mass (Table 1). Both species increased dry mass highly significantly with time, especially between harvests 1 and 2, but leaf dry mass (DML) decreased for *Microberlinia* and stabilized for *Tetraberlinia* at the third harvest. Interactions between K and Mg resulted in very few, and then only marginally, significant effects on dry mass.

**Table 1:** Dry seedling mass (DM, g) and its allocation to parts for the two tree species *Microberlinia bisulcata* and *Tetraberlinia bifoliolata* grown in a K x Mg factorial fertilizer addition experiment at the Mana Nursery near Korup. Parts: L, leaves; S, stem; R, roots; T, total. The values in the table are back‐transformed covariate‐adjusted means (averaging across the levels of the other factors), and therefore parts will not sum exactly to totals. The statistic (Fisher's variance ratio, *F*) and its significance are shown for the three main factors only: of the four interaction terms, these were very rarely significant and are not shown apart from their number (*NIntS*)

Factor	Level	*Microberlinia*				*Tetraberlinia*			
		DML	DMS	DMR	DMT	DML	DMS	DMR	DMT
K	1	5.29^b^	9.40^b^	9.22^a^	24.24^b^	13.71^a′^	18.60^a^	15.39^a^	48.18^a^
	2	5.52^ab^	10.54^ab^	9.86^ab^	26.26^ab^	13.83^a′^	17.55^a^	14.45^ab^	46.34^ab^
	3	5.98^a^	11.83^a^	10.55^a^	28.73^a^	13.07^ab′^	15.58^b^	13.30^b^	42.44^bc^
	4	5.96^a^	11.07^a^	10.41^ab^	27.72^a^	12.52^b′^	14.70^b^	13.41^b^	40.98^c^
Mg	1	4.99^b^	8.22^b^	8.51^b^	21.89^b^	12.55^a^	15.04^b^	12.86^b′^	40.85^b^
	2	5.73^a^	11.29^a^	10.36^a^	27.74^a^	13.34^a^	17.20^a^	14.73^a′^	45.79^a^
	3	6.24^a^	12.00^a^	10.94^a^	29.58^a^	13.77^a^	17.46^a^	14.79^a′^	46.43^a^
	4	5.84^a^	11.65^a^	10.36^a^	28.20^a^	13.41^a^	16.54^ab^	14.17^ab′^	44.66^ab^
Harvest	1	4.63^b^	5.94^c^	6.20^c^	16.84^c^	10.45^b^	10.01^c^	9.21^c^	29.82^c^
	2	6.47^a^	12.88^b^	11.39^b^	30.97^b^	14.53^a^	17.69^b^	15.32^b^	47.85^b^
	3	6.12^a^	15.89^a^	14.13^a^	36.45^a^	15.38^a^	25.51^a^	19.95^a^	61.25^a^
*F*‐values	K	1.71^ns^	3.71*	1.56^ns^	2.68*	2.04^ns^	7.37***	3.68*	5.27**
	Mg	8.05***	15.35***	7.06***	12.07***	1.52^ns^	2.59^o^	3.04*	2.88*
	Harvest	38.6***	176.2***	139.5***	145.6*	57.3***	178.9***	152.5***	159.0***
	*NIntS*	1	1	0	1	0	0	0	0

Means that do not share the same superscripted small letters among levels of the same factor are significantly different (*P *≤* *0.05). The ′‐marks to sets of letters indicate that differences are strictly insufficient as *P*(*F*) was > 0.05. [Error df: Mb, 137; Tb, 140.] Significance levels, *P*(*F*): ***≤0.001; **≤0.01; *0.05; ^o^≤0.10; ns >0.10.

**Figure 2:**
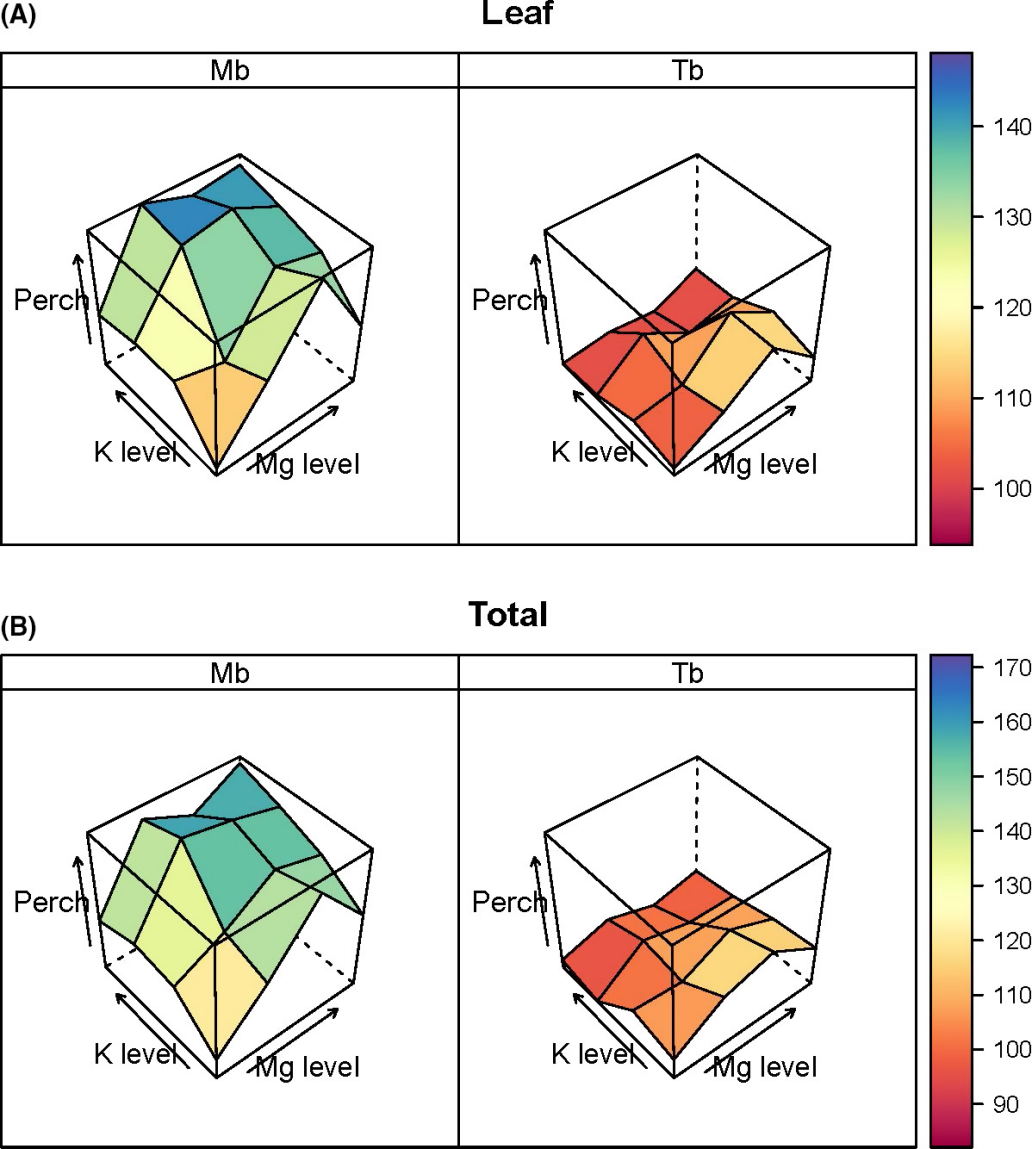
Percentage change (*perch*) in dry mass of the two species *Microberlinia bisulcata* (Mb) and *Tetraberlinia bifoliolata* (Tb) for the 16 treatments (four levels of K x four levels for Mg; each factor being null and three increasing additions), averaged across the three harvests, with reference to the common control [1,1] for (A) Leaf, and (B) Total, parts. (A value of ‘100 + *x*%’ would mean an *x*% increase over the control.) See App. Fig. 1 for *perch* in root and stem parts corresponding.

For any particular growth variable, each of the mean responses to the 16 treatment combinations (over the three harvests) could be related to the control as a percentage change (perch = {DM [*i,j*] / DM [1,1]} × 100, where *i* and *j* are levels of K and Mg factors, respectively). These means were the back‐transformed values from ANOVAs that incorporated shock as a covariate (i.e., they were different from means of untransformed and unadjusted means). Figure 2 highlights the marked difference between *Microberlinia* and *Tetraberlinia*, the former's response being much stronger than the latter for leaf and total dry mass, and with similar patterns for root and stem parts (Fig. S2). A large portion of the change was between L1 and L2, especially for Mg. Highest levels of application led to growth reductions, with maxima around L3 for both K and Mg. *Tetraberlinia* responded weakly to Mg, and barely to K, addition: *Microberlinia* responded strongly to Mg and partially to K addition, suggesting that only *Microberlinia* was Mg‐limited, and its positive response to Mg enabled it to respond further to the added K, that is, once Mg limitation was released, K limitation came into play.

The mean proportion of dry mass allocated to roots (DMR/DMT) was 0.376 for *Microberlinia* (root–shoot ratio, (DMR / [DML + DMS]) = 0.609) and correspondingly 0.320 (0.475) for *Tetraberlinia*. Only for *Microberlinia* across levels of Mg addition did the proportion alter with treatment, and as a significant decrease (0.390 to 0.369, L1 to L4; *P *<* *0.05). Between harvests 1 and 3, the ratio did increase slightly (*Microberlinia*: 0.370 to 0.389, *P *<* *0.001; *Tetraberlinia* 0.310 to 0.328, *P *<* *0.05). Leaf mass–area and height–diameter ratios were unaffected by the treatments (*P *>* *0.05). The effects of Mg and K addition on dry mass allocation were therefore very small for these two tree species' seedlings.

### Nutrient responses

Increasing levels of K significantly raised concentrations on K in the leaves of both *Microberlinia* and *Tetraberlinia*, again largely due to a difference between the control (L1) and the first addition level (L2), and likewise and even stronger was the effect of Mg addition on the concentration of leaf Mg (Table 2). However, added K led to *decreases* in leaf Mg concentration in both species, significantly for *Tetraberlinia* but less so for *Microberlinia*. Concentrations of Ca significantly decreased, respectively increased, with raised additions of K and Mg, in both species, whilst N concentration did not differ significantly across treatments for either species (Table 2). More marked was that K addition led to significant increases in leaf P concentration in both species too. As with the results for growth and dry mass responses, interactions between K and Mg were rarely or only weakly significant for concentrations, except for K addition on *Tetraberlinia*. For all elements and both species, leaf concentrations decreased over successive harvests (Table 2).

**Table 2:** Concentrations of macronutrients (mg/g) in leaves of seedlings for the two tree species *Microberlinia bisulcata* and *Tetraberlinia bifoliolata* grown in a K x Mg factorial fertilizer addition experiment at the Mana Nursery near Korup. The values in the table are back‐transformed covariate‐adjusted means (averaging across the levels of the other factors). The statistic (Fisher's variance ratio, *F*) and its significance are shown for the three main factors only: of the four interaction terms, these were not often significant (and only then at *P *<* *0.01) and are not shown apart from their number (*NIntS*)

Factor	Level	*Microberlinia*					*Tetraberlinia*				
		Ca	K	Mg	N	P	Ca	K	Mg	N	P
K	1	7.29^a^	5.73^c^	4.78^a′^	19.29^a^	0.849^b^	5.47^a^	3.75^c^	2.66^a^	14.70^a^	0.648^b^
	2	6.49^b^	7.55^b^	4.69^ab′^	19.40^a^	0.850^b^	4.55^b^	5.48^b^	2.16^b^	14.73^a^	0.655^b^
	3	5.90^c^	8.40^ab^	4.70^ab′^	19.22^a^	0.918^b^	4.51^b^	6.50^a^	1.77^c^	14.91^a^	0.715^a^
	4	6.08^bc^	8.85^a^	4.58^b′^	19.65^a^	1.017^a^	4.53^b^	6.44^a^	1.98^bc^	15.26^a^	0.730^a^
Mg	1	5.65^c^	10.20^a^	1.41^c^	20.74^a^	0.992^a^	4.24^c^	5.94^a^	0.99^c^	14.95^a^	0.698^a^
	2	6.42^b^	6.89^b^	6.19^b^	19.10^b^	0.853^b^	4.50^bc^	5.38^b^	2.47^b^	14.83^a^	0.675^a^
	3	6.51^b^	6.90^b^	7.41^a^	18.92^b^	0.901^b^	4.89^b^	5.13^b^	2.73^ab^	15.07^a^	0.685^a^
	4	7.19^a^	6.63^b^	7.45^a^	18.86^b^	0.882^b^	5.45^a^	5.25^b^	3.00^a^	14.74^a^	0.687^a^
Harvest	1	7.69^a^	9.11^a^	5.29^a^	22.52^a^	1.065^a^	5.76^a^	6.35^a^	2.22^a^	17.74^a^	0.884^a^
	2	6.44^b^	7.03^b^	4.58^ab^	18.24^b^	0.792^c^	4.36^b^	5.31^b^	2.12^ab^	14.15^b^	0.621^b^
	3	5.34^c^	6.65^b^	4.24^b^	17.74^c^	0.880^b^	4.26^b^	4.72^c^	2.01^b^	13.18^c^	0.588^b^
*F*‐value	K	6.66***	15.49***	0.38^ns^	0.27^ns^	2.76*	8.01***	94.96***	18.38***	1.37^ns^	5.69***
	Mg	10.91***	31.87***	172.17***	5.55***	3.98**	10.72***	5.86**	162.41***	0.43^ns^	0.26^ns^
	Harvest	49.1***	30.0***	4.3*	66.9***	31.2***	33.9***	41.7***	2.1^ns^	144.2***	98.0***
	*NIntS*	1	2	2	0	0	2	3	2	0	1

Means that do not share the same superscripted small letters among levels of the same factor are significantly different (*P *≤* *0.05).The ′‐marks to sets of letters indicate that differences are strictly insufficient as *P*(*F*) was > 0.05. [Error df: Mb, 129; Tb, 133.] Significance levels, *P*(*F*): ***≤0.001; **≤0.01; *0.05; ^o^≤0.10; ns >0.10.

**Figure 3:**
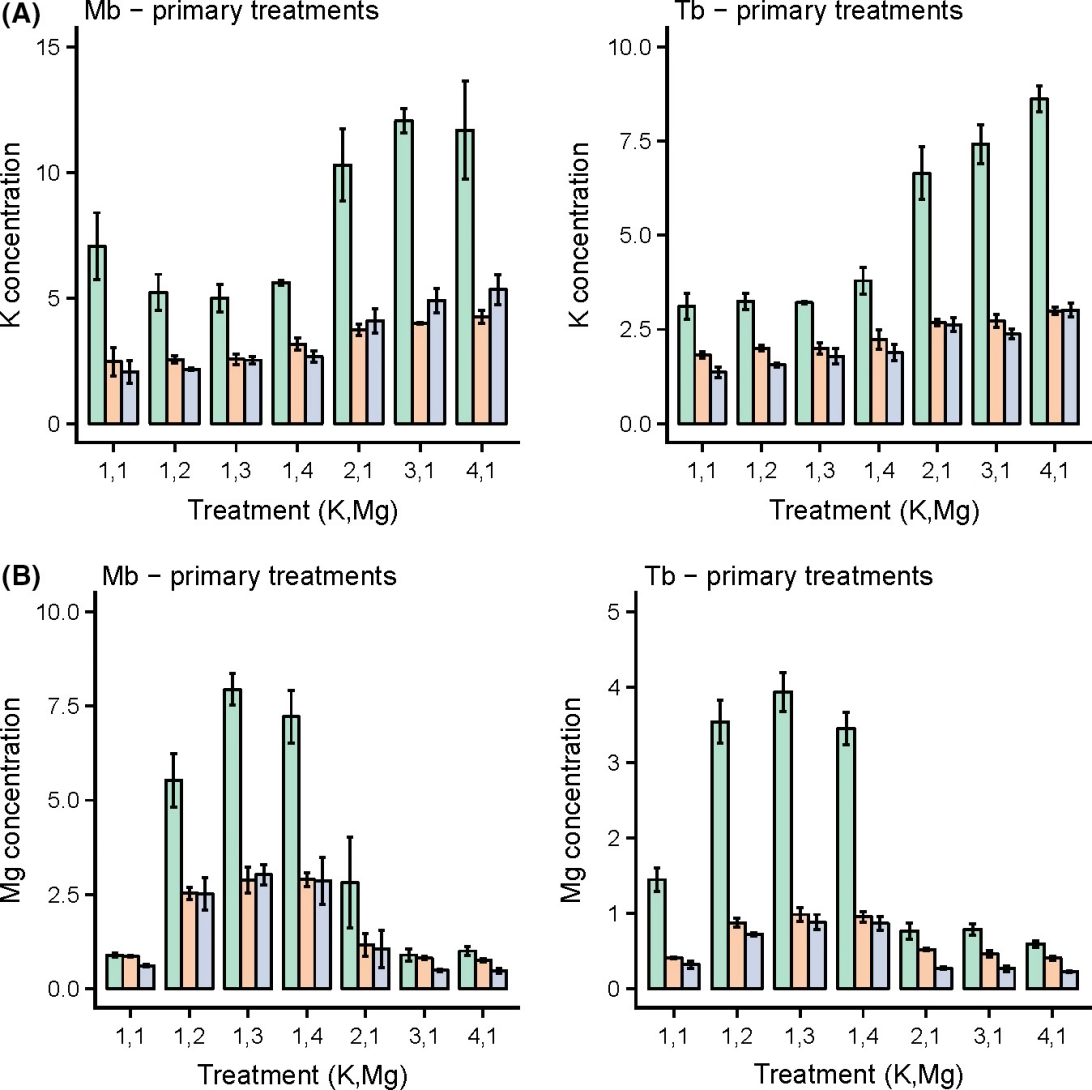
Mean concentrations (mg/g) of (A) Potassium, and (B) Magnesium, in leaves (green), roots (brown), and stems (light blue bars) of seedlings of *Microberlinia bisulcata* (Mb) and *Tetraberlinia bifoliolata* (Tb) at harvest 2 for the primary treatments. A primary treatment had at least one factor at level 1. Concentrations for the corresponding secondary treatments (no factor at level 1) are shown in App. Fig. 2.

Addition of K and Mg had corresponding effects of significantly increasing K and Mg concentrations, respectively, also in roots and stems of both species (harvest 2, Tables S2a, b), but there were hardly any other significant effects of these additions on concentrations of the other elements, besides K addition increasing P concentrations in both species (three of four cases at *P *≤* *0.05). Mg addition significantly increased concentrations of Ca in roots of both species, however (Table S2b). As with leaves, the main differences were between the controls (L1) and first addition levels (L2). At harvest 2 – by then seedlings were almost completely free of shock effects – grouping into those primary (Fig. 3) and those secondary (Fig. S3) indicated how across the 16 treatments, Mg and K concentrations were varying far less than in the leaves, although still largely being correlated positively with trends in the latter. The higher levels of K addition led to rather smooth or similar sets of responses in their K and Mg concentrations for *Microberlinia* plant parts, although in comparison responses were less consistent for *Tetraberlinia* (Fig. 3). By contrast, Mg addition resulted in clearer, more pronounced, changes in both elements, especially for the leaves of *Microberlinia*. For instance, where Mg was added with K, this led to sustained high concentrations of Mg, but not of K, for *Microberlinia* at the higher treatment combinations (Fig. S3, secondary treatments). This was not quite the case for *Tetraberlinia*, with Mg concentrations *decreasing* when combined with K addition, and conversely for this species added Mg sustained the high K concentrations. Although interactions were not always strong, there is some evidence of a reciprocal effect: K enhancing Mg for *Microberlinia* and vice versa for *Tetraberlinia*.

**Figure 4:**
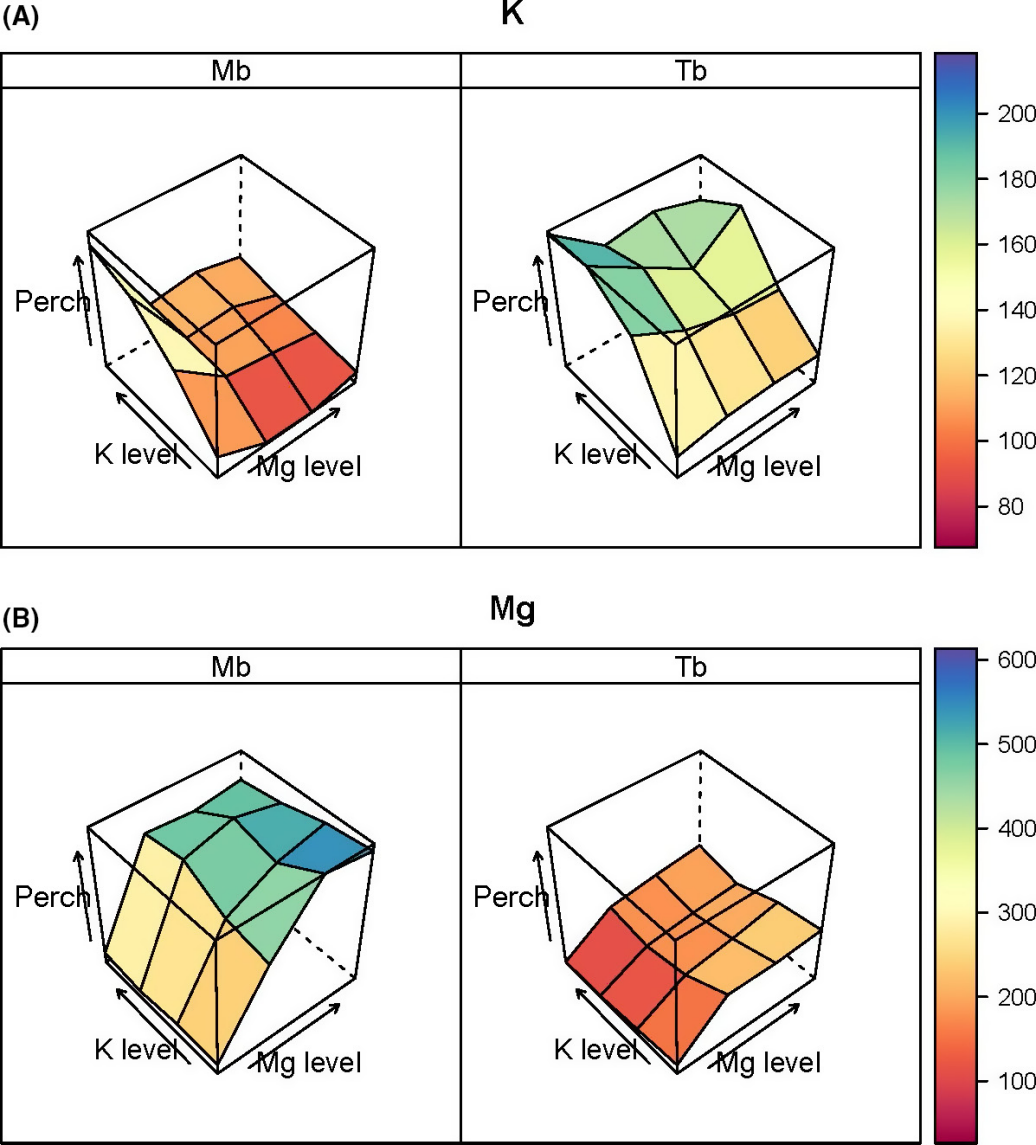
Percentage change (*perch*) in (A) Potassium, and (B) Magnesium, leaf nutrient concentrations of *Microberlinia bisulcata* (Mb) and *Tetraberlinia bifoliolata* (Tb) for the 16 treatments with reference to the common control. Explanations of factor levels and *perch* are given in Fig. 2.

Differences in Ca were likewise better structured for *Microberlinia* than *Tetraberlinia* (Fig. S4A), yet barely existing for N and P, for either species (Fig. S4B and C). However, a clear difference between N and P vs. K, Mg, and Ca was that the former group had higher concentrations in roots than stems, and these were closer to corresponding leaf concentrations, than did the latter group. For *Tetraberlinia*, the closest leaf, root, and stem concentrations occurred for P at the higher levels of K and Mg addition, pointing to K perhaps aiding P uptake for this species, which was not the case for *Microberlinia*. Percent changes in K and Mg concentrations in leaves were also very different between species, suggesting further a reciprocal pattern of response with *Microberlinia* taking up (and responding to) more Mg as it was applied and responding less to K; and *Tetraberlinia* responding more to K addition and less to Mg (Fig. 4). Percentage changes in Ca, N, and P were much less well defined with respect to K and Mg additions (Fig. S5).

### Soil–seedling relationships

Additions of K and Mg as treatments had the expected effects on the available concentrations of these elements in the pot soils (Tables S3a, b). For K addition, pot concentrations were approximately linearly related to the amounts added, and for Mg addition, these were more convexly curvilinear (the change from control [L1] to first level added [L2], being greater than L2 to L3/L4). The second fertilizer application must clearly have raised the concentrations in the pots considerably at harvest 2 for this to be reflected in leaf concentrations. In the upper parts of the pots, Ca concentrations increased with application levels for both species, but significantly so only when Mg was added. The lower parts of the pots showed the opposite effect for Mg addition. Nevertheless, Mg addition also decreased soil K concentrations for both species in both upper and lower soils. The effect of K addition on Mg concentration was seemingly complex due to many interactions between the main factors, species, and pot parts: for *Microberlinia*, it moderately increased Mg in the upper soil, but in *Tetraberlinia*, it strongly decreased it in the lower soils (Tables S3a, b). The response surfaces showed how Mg addition affected less the K levels as K addition affected the Mg levels (Fig. S6A and B).

Means of upper‐ and lower‐layer concentrations of K and Mg in pot soils were significantly positively (and linearly) dependent on the level of fertilizer applied for both species by harvest 1 (*P *<* *0.001), confirming the efficacy of the treatments overall with slopes of 11.9 and 11.6 *μ*g/g K for *Microberlinia* and *Tetraberlinia*, respectively, and correspondingly 19.1 and 23.2 *μ*g/g Mg. As each incremented level of addition was equal to 2.08 g_a_ K and 0.83 g_a_ Mg per pot (start to harvest 1; g_a_ means g‐applied), then averaging over species' pot concentrations, the treatment efficacy was 5.65 *μ*g/g/g_a_ K and 51.0 *μ*g/g/g_a_ Mg. Thus, Mg was made much more available than K in the soil for the seedlings. Furthermore, regressions had very different slopes for K depending on whether upper or lower pot soils were considered, with the slopes of 2.80 and 3.39 *μ*g/g, for *Microberlinia* and *Tetraberlinia*, respectively, for upper soils; and 21.1 and 19.8 *μ*g/g for lower ones. By contrast, the slopes for Mg were closer between layers with corresponding values 14.6 and 16.9, and 23.5 and 29.4 *μ*g/g. Concentrations across blocks were consistent for each element. These results indicate that strong leaching occurred down the pot profile for K, and moderate leaching for Mg.

Generalized linear model fits for *Microberlinia* total seedling dry mass (DMT) at harvest 1 against K and Mg level number (L1‐L4, i.e., exactly linearly related to the application rates) were slightly better for Mg (Mg, *P *=* *0.025; K, *P *=* *0.84) than when the actual mean pot concentrations were used as independent variables (Mg, *P *=* *0.054; K, *P *=* *0.40). And in a similar way K fits were better too with the original level numbers (K, *P *=* *0.004; Mg, *P *=* *0.096) than the actual concentrations (K, *P *=* *0.049; Mg, *P *=* *0.12). Thus, whilst confirming the main results of analyses of variance based on levels applied, more detailed knowledge of the pot concentrations led to weaker rejection of the key null hypotheses (Mg effect for *Microberlinia*, K effect for *Tetraberlinia*).

Concentrations of K and Mg in leaves were strongly and significantly positively correlated with those in roots and shoots for both species, and likewise with the corresponding pot concentrations (Table 3). For Ca, N, and P, correlations were far weaker and mostly not significant. Total dry mass (DMT) correlated negatively with leaf K (significantly for *Tetraberlinia*,* P *<* *0.01; not for *Microberlinia*), strongly positively with leaf Mg (*P *<* *0.001, both species significant), but weakly and marginally with Ca, N, and P concentrations (*P *<* *0.10). Usually K, P, and N were positively intercorrelated as were K, Ca, and Mg (Table 3), although more essential was that Mg and N were strongly and significantly *negatively* correlated for *Microberlinia* yet not for *Tetraberlinia*.

**Table 3:** For *Microberlinia bisulcata* (Mb) and *Tetraberlinia bifoliolata* (Tb), correlations (Pearson's *r*), across the *n *=* *16 treatment combination means, between *ln*‐transformed nutrient concentrations in leaves (L) vs. those in stems (S) and roots (R), total seedling dry mass (DMT), the same nutrients' concentrations in pot soils (U = upper, L = lower), and other nutrients' leaf concentrations. No measurements made are indicated by “–”

	L vs. S	L vs. R	L vs. DMT	L vs. Soil_U_	L vs. Soil_L_	L vs. L			
Mb						K	Mg	Ca	N
K	0.837***	0.806***	−0.290^ns^	0.855***	0.762***				
Mg	0.986***	0.963***	0.816***	0.930***	0.958***	−0.701**			
Ca	−0.047^ns^	−0.379^ns^	0.186^ns^	0.448^o^	−0.182^ns^	−0.886***	0.630**		
N	−0.003^ns^	0.365^ns^	−0.582*	–	–	0.667**	−0.777***	−0.582*	
P	0.605*	0.406^ns^	−0.124^ns^	–	–	0.758***	−0.454^o^	−0.675**	0.568*
Tb									
K	0.850***	0.910***	−0.668**	0.862***	0.968***				
Mg	0.968***	0.919***	0.646**	0.872***	0.953***	−0.508*			
Ca	0.208^ns^	−0.316^ns^	0.489^o^	0.149^ns^	−0.211^ns^	−0.716**	0.683**		
N	0.142^ns^	0.236^ns^	−0.226^ns^	–	–	0.464^o^	−0.128^ns^	−0.278^ns^	
P	0.793***	0.590*	−0.442^o^	–	–	0.703**	−0.307^ns^	−0.402^ns^	0.681**

***P ≤* *0.001; **P ≤* *0.01; *P ≤* *0.05; ^o^ *P* ≤ 0.1; ^ns^ *P *>* *0.1.

Mean pot concentrations across species in controls (L1) at harvest 1 were 15.6 *μ*g/g K and 9.4 *μ*g/g Mg, and raising each to addition level 1 (L2) resulted in 44.7 *μ*g/g K and 44.3 *μ*g/g Mg. In the preparation of the pot medium, the top 6 cm of soil at Isangele Road had been taken and mixed. In separate soil sampling for two related experiments performed in March 2012, one at Isangele Road and the other in main P‐plot in Korup, the top 6 cm had weighted mean available concentrations of 44.4 and 45.8 *μ*g/g K and 21.2 and 25.4 *μ*g/g Mg. This means that by harvest 1, plants in the controls had depleted the available K and Mg concentrations, and level 1 addition had reinstated it for K but overstated it for Mg.

## Discussion

### Nutrient levels and demands

Experiments of a similar type elsewhere in the tropics for primary forests are very few and far between, and only two by Burslem et al. (1995, 1996) added Mg as one of their single treatments to a native soil. In a third experiment by Gunatilleke et al. (1997), Mg was added too but to a non‐native soil and factors Mg and P were partially confounded. In the experiment of Turner et al. (1993), K was part of a general NPK treatment and therefore its effect not readily separable from that of P and N, although in two other studies K was added singly (Nilus et al. 2011; Santiago et al. 2012). Nutrient supply to seedlings of very different species, from Sri Lanka, SE Asia, and Central America, is unlikely to be of too much ecological relevance for the very different species and soils at Korup though. Because of the widely assumed but unfounded notion of nutrients being nonlimiting for tropical rain forest tree seedlings, although possibly limiting tree growth (Wright et al. 2011 for K in particular), it is perhaps not surprising that there are only just two studies published for Mg, and so very few others involving K. Further reported experiments compared different soils and their nutrient levels on seedling growth using correlative approaches (e.g., Juliana et al. 2009; Holste et al. 2011), but these at best can only be indicative.

Whilst seedlings of *Microberlinia* and *Tetraberlinia* grown in the control Korup soils appear to have been differentially limited in their growth by Mg and K supply, they were not severely deficient in either element. This is evidenced by the absence of characteristic leaf deficiency symptoms, especially the chlorotic leaf tissues characteristic of plants with very low Mg or necrosis at leaf tips and edges when K is critically low (Marschner 1995; Barker and Pilbeam 2007), and by the fact that very few plants died during the experimental period, even among the controls. Furthermore, there was little experimental evidence of large changes in R/S ratio (*c*. 5% only for *Microberlinia*) with increasing supply of Mg and K, a phenomenon shown repeatedly for herbaceous and crop plants, although only when the elemental levels were very severely reduced (Hermans et al. 2005, 2006). There was no evidence of fungal attack, which is often associated with a deficiency of K (Marschner 1995). Nevertheless, some *Microberlinia* seedlings did lose leaflets across all treatments over time, and this loss was usually proceeded by a yellowing of the leaves: the analysis of mean individual LA per plant did not suggest any linkage with control vs. additions though. Deficiencies may have been obscured on some plants accordingly and gone unrecorded because individual leaflets are so small.

For 21 tree species' values taken from the extensive tabulation of Merhaut (2007), mean minima and mean maxima Mg concentrations defined diagnostically as being ‘sufficient’ in leaves (with SEs and ranges) were 1.86 ± 0.15 mg/g (0.9–3.5) and 7.09 ± 0.51 mg/g (3.6–12.0). The two species' values in Table 2 sit well within this range except for the controls (Mg L1) being both definitely lower, and on this basis can be labeled ‘insufficient’. More general data for K suggested that typically sufficient concentrations lie in the ranges 6–10 mg/g for conifers, 11–20 mg/g for fruit trees, and 12–15 mg/g for broad‐leaf trees (all temperate; Mengel 2007). In this respect, all values in Table 3 are low, even though the concentrations were close to the ranges for other Korup experiments and no strong effect of added K was found. Are rain forest tree seedlings inherently low in K? Burslem et al. (1996) and Nilus et al. (2011) reported K leaf concentrations in the range 10–15 mg/g for nursery seedlings of five rainforests tree species, which points to the situation in Korup being rather special with wildings and out‐plants reaching just 10 mg/g K (Table 4). In an extensive 8‐year field experiment in Panama investigating limitations of N, P, and K, Santiago et al. (2012) found that adding K had a small but significant effect on in situ seedling growth of five understory species. Concentrations of leaf K and Mg ranged from 3.96 to 35.16 mg/g and 0.77 to 8.41 mg/g, respectively (L. Santiago, pers. comm.).

**Table 4:** Comparison of K and Mg concentrations (mg/g) in leaves of *M. bisulcata* (Mb) and *T. bifoliolata* (Tb) seedlings in this and previous experiments. L1 and L3 are the first and third addition levels of K and Mg when the other element was not added. “–” indicates that no data were available. Experimental values are expressed as percentages (%) of the averages of the other studies’ values

	K				Mg			
	Mb	(%)	Tb	(%)	Mb	(%)	Tb	(%)
This experiment – Control	6.71	82	3.55	47	1.45	45	1.31	68
L1	9.96	121	6.42	85	5.38	168	3.32	172
L3	13.32	162	7.34	97	8.01	250	3.46	179
Other studies:								
Light quantity trial^a^	5.91		7.24		2.57		2.24	
Transplants – P‐plot^b^	9.55		8.13		2.56		1.77	
P‐fertilization trial^b^	3.90		–		3.11		–	
Wildings in P‐plot^c^	10.99		–		4.19		–	
Herbivory trial^c^	9.78		7.32		3.99		1.78	
Nursery out‐plants^d^	9.04		–		2.86		–	
Average, others	8.20		7.56		3.21		1.93	

Green and Newbery (2001a).

a Green and Newbery (2001b).

b Newbery et al. (2002).

Unpublished data (D. M. Newbery).

c Norghauer et al. (2014).

d Schwan (2003).

Note: the ECM infection trial of Newbery et al. (2000) was excluded here as the roots had been initially sterilized.

The comparison with leaf concentrations in other Korup studies, experimental and field, indicated that Mg (in controls, L1) was suboptimal for both *Microberlinia* and *Tetraberlinia*, rising toward field levels under Mg addition, whilst for K there was no clear case of suboptimality for *Microberlinia* (concentrations on par with other experiments and field studies), although for *Tetraberlinia* there was a stronger difference (Table 4). This comparison suggests that *Microberlinia* is strongly Mg‐ but not K‐limited, and *Tetraberlinia* neither K‐ nor Mg‐limited, in the Korup soil. But it must be remembered that the seedlings in the present experiment, although using freshly collected soils from the forest, were, as in all such similar local nursery ones, growing ex situ. The shock resulting from the first addition of the higher levels of K for *Microberlinia*, but not *Tetraberlinia*, was most pronounced among blocks with the smaller seedlings. The phenomenon can be explained by *Microberlinia* tending to have more fine roots near the soil surface than *Tetraberlinia* (G. Neba, pers. obs.), and thus the temporarily high K concentrations in the top 1–2 cm of the pots would probably have invoked a strong local osmotic effect after first watering.

### Response physiology: K and Mg

The addition of Mg had significant effects on the growth and dry mass in both species. Seedlings that received additions of this element grew better in terms of height, diameter, and leaf number than those without it. They produced correspondingly larger leaf areas and more dry matter for the different parts than control seedlings. The effects of Mg on *Microberlinia* were far more pronounced than on *Tetraberlinia*: for both species different plant parts changed in similar proportions. It can be concluded that Mg was limiting nursery growth in *Microberlinia*, and probably does so too in the forest. In a similar way, *Tetraberlinia* appears not to be K‐limited. Nevertheless, a proviso is needed because the K leaf concentrations in *Microberlinia* and *Tetraberlinia* at Korup are relatively low; and so it is curious that adding plenty of K experimentally did not raise them much higher. Interestingly, mature canopy leaves of *Microberlinia* and *Tetraberlinia* also had relatively low K concentrations of 5.91 and 4.23 mg/g, respectively (Chuyong et al. 2000).

Mg addition has been shown generally to increase chlorophyll content, phloem loading, and transport of photosynthates to sink sites and thus affect productivity (Marschner 1995). In the herbaceous crop plants *Phaseolus vulgaris* and *Beta vulgaris*, transport of sucrose out of leaves can be restricted under severe Mg‐deficiency (Cakmak et al. 1994a,b), leading to accumulations fourfold those of Mg‐sufficient leaves (Hermans et al. 2004, 2005). Strong Mg‐deficiency can inhibit root development, especially fine root growth (Marschner 1995; Ericsson and Kahr 1995; for *Betula pendula*; McDonald et al. 1996; for *Alnus incana*; Hermans et al. 2006; review). Given that Mg was most likely not so severely deficient in the present experiment, the most obvious role for it was building chlorophyll a and b, and hence improving the rate of photosynthesis (Laing et al. 2000 for *Pinus radiata;* Mehne‐Jakobs 1995, 1996 for *Picea abies*; Yang et al. 2012; for *Citrus* spp.; Cakmak and Kirkby 2008; review). Mg bound to H^+^‐ATPase also effects several plasma‐membrane processes, is a constituent of many protein‐building enzymes, and is further involved in photosynthetic reactions (Epstein and Bloom 2005; Yang et al. 2007;). Despite the known role of K in stomatal regulation, osmotic potential in the vacuoles, and translocation physiology, it is not so directly involved in photosynthesis (Marschner 1995). These findings are largely for temperate herbaceous and woody (mostly conifer) plants, and whilst investigations on tropical tree seedling physiology regarding K and Mg are almost entirely lacking, the basic plant physiology is expected to be generally applicable.

Nutrient limitation in seedlings was indicated by nutrient concentrations in the various plant parts, especially leaves (Marschner 1995), and by percentage change in concentration across addition levels from the control to the treatment showing the highest plant dry mass. The mean percentage changes (*perch*) of 526 and 276% at Mg level 3 (2nd addition level; Tables 1 & 2) in leaf Mg concentration for *Microberlinia* and *Tetraberlini*a, respectively, support the idea that Mg was causing the increases in dry mass with lower additions, effects being much stronger for *Microberlinia* than *Tetraberlini*a (Fig. 4), but later inevitably some other factor would be limiting growth when Mg came into luxury supply. Increasing K addition had little further influence for either species' change in Mg concentration. Very differently, however, at this Mg level leading to the maximum seedling mass, increasing K addition had barely any effect on K leaf concentrations for *Microberlinia,* whilst for *Tetraberlinia* increasing it did increase K concentrations (Fig. 4). One interpretation might be that *Microberlinia* is not K‐limited when Mg is sufficiently or optimally available, whilst *Tetraberlinia* is apparently moderately Mg‐limited when K is sufficiently or optimally available. This emphasizes a basic set of reciprocal responses by the two species, to additions of Mg and K, as a complex and interesting nonlinear asymmetrical form of interaction. Moreover, there is no strong evidence in this experiment for ionic antagonism between K and Mg as demonstrated by Sun and Payn (1999) for *Pinus radiata* and Ding et al. (2006) for *Oryza sativa*. This was probably because there was no strong deficiency in either element, and this was shown too by the relative changes in their plant parts concentrations, as the other factor was increasingly applied, being very similar.

For Ca, N, and P, percentage change values of <100 showed that the fast growth induced by Mg addition used up and diluted the concentrations of these other elements. Such dilution effects also occurred as K addition was increased, although less strongly. This may also point to their limiting growth at the optimal level of Mg application, which was again stronger for *Microberlinia* than *Tetraberlinia*. Addition of small amounts of Ca, N, and P together with Mg at level 3 would be expected to further improve the growth response of *Microberlinia*, but not necessarily that of *Tetraberlini*a. For the present experiment, based on the intraleaf concentration (negative) correlations in Table 3, the sequences Mg ‐> N ‐> P for *Microberlinia*, and K ‐> Mg ‐> Ca for *Tetraberlinia* suggest the ordering of relevant limiting factors. The results here concur with Rubio et al. (2003) that trying to decide between a ‘law of the minimum’ sensu Liebig and a multiple limitation hypothesis is not too useful, and each element or factor should be examined individually for its effects and interactions with others, e. g., the complex interactions between Mg and K (then P).

### Interactions with other elements

Although leaf P concentrations varied little across treatments in this K‐ and Mg‐addition experiment, they were lower for *Microberlinia* and *Tetraberlinia*, at 0.91 and 0.69 *μ*g/g, respectively, than controls in the previous nursery and P‐addition trial (Green and Newbery 2001a; Newbery et al. 2002) and averages of the two out‐planting studies (Newbery et al. 2000; Green and Newbery 2001b) involving both species at Korup): four‐studies means of 1.66 *μ*g/g (range 1.31–2.28 *μ*g/g) and 1.20 *μ*g/g (range 1.10–1.27 *μ*g/g), respectively, that is, 55 and 58% the earlier values. Leaf N concentrations were also lower but less so: in the present experiment 19.4 and 14.9 *μ*g/g for the two species, respectively, compared with four‐studies means of 24.2 (19.5–28.3) *μ*g/g and 19.5 (15.6–22.4) *μ*g/g; correspondingly 80% and 76%. This would suggest that P availability in the experiment was suboptimal and partially limiting to all plants, making the implied increase in uptake of P by increased additions of K for *Microberlinia* and *Tetraberlinia* (Table 4) especially interesting. Any P‐limitation was being partly mitigated by added K. Nevertheless, the two previous experiments that tested for P‐limitation directly (i.e., by adding P) found no evidence for it (Green and Newbery 2001a; Newbery et al. 2002).

Magnesium may have a different, possibly more important, effect on plant growth from being just a required nutrient. One adaptive mechanism to Al toxicity in acid soils is the release of organic acid anions from roots, and for this Mg is required (Yang et al. 2007; Bose et al. 2011; Brunner and Sperisen 2013). Organic acid anions chelate Al cations by forming nonphytotoxic Al‐organic acid complexes (Marschner 1995; Miyasaka et al. 2007). The tropical soils of Korup are sandy, leached, and acid (pH 4.0‐5.8; Gartlan et al. 1986) and almost certainly (although not, unfortunately, so far confirmed by measurement) high in Al (see Hawkins and Brunt 1965 for the region in general). The element Al competes more actively with Mg at root uptake sites and in metabolic processes than with K (and Ca), because the first two elements have much more similar radii than the others (Miyasaka et al. 2007). This means that interactions are more likely between Al and Mg, not between Al and K. So not only does Al lead to reduced uptake of Mg into plants and interferes with metabolism where Mg is involved, and some exudation of organic acids is induced by the Al alone as a tolerance mechanism by many species, the addition of Mg will raise organic acid production further, thereby ameliorating the toxicity and allowing sufficient uptake and utilization of Mg with the exclusion of Al in the soil. For *Pinus radiata* in New Zealand, Mitchell et al. (1999) not only showed that adding Mg fertilizer reduced canopy Mg‐deficiency symptoms but also lowered exchangeable soil Al concentrations. For tropical tree seedlings, Burslem et al. (1996) seem to have been the first to hint at a link between Mg and Al in acid soils, albeit for an Al‐accumulating species, *Antidesma cuspidatum*.

This subtle interplay between Al and Mg concentrations at the root surface may in part explain why the two species, *Microberlinia* especially, continued to grow well above Mg addition level 1 (L2), which would have removed its limitation as such. The high tissue concentrations of Mg with higher Mg additions were probably a reflection of the beneficial effects of removing the putative Al toxicity. It would suggest, as a competing hypothesis to *Microberlinia* being more Mg‐limited than *Tetraberlinia*, that *Microberlinia* is much more sensitive to Al toxicity than *Tetraberlinia*, and hence Mg availability both supplies and ameliorates. There is some evidence that ectomycorrhizas can aid Mg uptake by trees (Jentschke et al. 2000, 2001; for *Picea abies*) but whether they can ameliorate Al toxicity remains still inconclusive (Marschner 1995; Miyasaka et al. 2007). Ahonen‐Jonnarth et al. (2000) and Tahara et al. (2005) showed, however, that increased Al concentrations around roots (of *Pinus sylvestris* and *P. densiflora*) led to greater exudation of organic acids when seedlings were ectomycorrhizal as opposed to nonectomycorrhizal. In the present experiment, it appears that ectomycorrhizas, at least for *Microberlinia*, had a relatively small influence on treatment effects, and probably for *Tetraberlinia* too.

### Ecological aspects: recruitment

As a plant‐growth study, nursery conditions allowed control of many other interacting factors that would occur in the forest (e.g., light level was fixed and the same across all treatments). More importantly, the influence of the ectomycorrhizal mat was excluded. Knowing the intrinsic physiological responses of the two tree species allows an assessment of processes that might be exacerbating or alleviating nutrient limitation in the field (e.g., soil leaching vs. mat retention and nutrient supply to seedlings, G. A. Neba, unpublished data). The experiment reveals only in part, but clearly reflects, the regeneration niches of the two species. Certainly, other factors will define their full niches in the field.

*Microberlinia* and *Tetraberlinia* responded quite differently to additions of K and Mg, suggesting that the former is relatively more Mg‐demanding and the latter more K‐demanding. Importantly, both were grown at moderately high light intensities in the Mana nursery, not the more common shady understorey conditions of the forest. A further caveat is that seedlings were already well established (>6 months old) when the treatments were first applied: possibly differences in response might have been larger had they been first treated earlier. *Microberlinia* is a very shade‐intolerant and strongly light‐responding species, whilst *Tetraberlinia* is shade‐tolerant and moderately light‐responding (Green and Newbery 2001a,b). Their differences in demands and responses may be simply a reflection of the basic physiologies, with Mg being primarily involved in photosynthesis in the light, K in storage and translocation in the shade. A more complex interpretation does not seem warranted at this stage. Whether both species are low‐K tolerant given their low tissue concentrations, the weak response to added K, and (besides being low‐P) the low K availability in the soil at Korup (Gartlan et al. 1986; Newbery et al. 1988, 1997), remains an open question.

If the K‐Mg availability ratio were to vary spatially in the forest, then a low value, coincident on a lighted area, would presumably be needed to enhance the chances of *Microberlinia* recruiting better than *Tetraberlinia*; or, conversely, if soil Mg concentrations can be shown to be very low around adult *Microberlinia* trees due to a high demand (Chuyong et al. 2000, 2002) for the element internally (to detoxify Al externally), then this limitation might in part explain the poor recruitment of *Microberlinia* seedlings in the Korup groves. Because Mg and K are both recycled via the mineralization of litterfall and canopy throughfall (Chuyong et al. 2002, 2004), species' growth responses to K and Mg need to be more closely linked in further studies to the relative nutrient inputs and availabilities in the soil. The reported experiment also demonstrates that, whilst it is difficult to completely understand how the causal nexus of interacting elemental concentrations affects tree seedling growth in the field, simplification under the more controlled conditions of a nursery does give valuable insights and new testable hypotheses about the mechanisms involved.

## Data Accessibility

Data are available from the Dryad Digital Repository: http://dx.doi.org/10.5061/dryad.k7729.

## Conflict of Interest

None declared.

## Supporting information


**Figure S1.** *Microberlinia bisulcata* (Mb) and *Tetraberlinia bifoliolata* (Tb) growing within the shade house at the Mana Bridge Nursery, next to Korup, Ndian.

**Figure S2.** Percentage change (*perch*) in dry mass of the two species *Microberlinia bisulcata* (Mb) and *Tetraberlinia bifoliolata* (Tb) for the 16 treatments (four levels of K x four levels for Mg; each factor being null and three increasing additions), averaged across the three harvests, with reference to the common control [1,1] for (a) root, and (b) shoot parts. This complements leaf and total parts in Fig. 2.

**Figure S3.** Mean concentrations (mg/g) of potassium and magnesium, in leaves (green), roots (brown) and stems (light blue bars) of seedlings of *Microberlinia bisulcata* (Mb) and *Tetraberlinia bifoliolata* (Tb) at harvest 2 for the secondary treatments. A secondary treatment had no factor at level 1. Concentrations for corresponding primary treatments are shown in Fig. 3.

**Figure S4.** Mean concentrations (mg/g) of (a) calcium, (b) nitrogen, and (c) phosphorus, in leaves (green), roots (brown) and stems (light blue bars) of seedlings of *Microberlinia bisulcata* (Mb) and *Tetraberlinia bifoliolata* (Tb) at harvest 2 for the primary and secondary treatments. A primary treatment had at least one factor at level 1, whilst secondary treatments had none.

**Figure S5.** Percentage change (*perch*) in calcium, nitrogen and phosphorus leaf nutrient concentrations of *Microberlinia bisulcata* (Mb) and *Tetraberlinia bifoliolata* (Tb) for the 16 treatments with reference to the common control. Explanations of factor levels and *perch* are given in Fig. 2.

**Figure S6.** Percentage change (*perch*) in (a) potassium, and (b) magnesium, concentrations in the upper and lower layers of the pot soil, for the 16 treatments with reference to the common control. Explanations of factor levels and *perch* are as for Fig. 2.

**Table S1.** Seedling sizes for the two tree species *Microberlinia bisulcata* and *Tetraberlinia bifoliolata* grown in a K x Mg factorial fertilizer addition experiment at the Mana Nursery near Korup.

**Table S2.** (a) Concentrations of macronutrients (mg/g) in stems at one harvest (H2) of seedlings for the two tree species *Microberlinia bisulcata* and *Tetraberlinia bifoliolata* grown in a K x Mg factorial fertilizer addition experiment at the Mana Nursery near Korup. (b) Concentrations of macronutrients (mg/g) in roots at one harvest (H2) of seedlings for the two tree species *Microberlinia bisulcata* and *Tetraberlinia bifoliolata* grown in a K x Mg factorial fertilizer addition experiment at the Mana Nursery near Korup.

**Table S3.** (a)Concentrations of three cations (*μ*g/g) in the upper soil layer of pots with seedlings of *Microberlinia bisulcata* and *Tetraberlinia bifoliolata* grown in the K x Mg factorial fertilizer addition experiment. (b) Concentrations of three cations (*μ*g/g) in the lower soil layer of pots with seedlings of *Microberlinia bisulcata* and *Tetraberlinia bifoliolata* grown in the K x Mg factorial fertilizer addition experiment.

## References

[ece31835-bib-0001] Ahonen‐Jonnarth, U. , P. A. W. Van Hees , U. S. Lundstrom , and R. D. Finlay . 2000 Organic acids produced by mycorrhizal *Pinus sylvestris* exposed to elevated aluminium and heavy metal concentrations. New Phytol. 146:557–567.

[ece31835-bib-0002] Barker, A. V. , and D. J. Pilbeam , eds. 2007 Handbook of plant nutrition. CRC: Taylor & Francis, Boca Raton.

[ece31835-bib-0003] Bose, J. , O. Babourina , and Z. Rengel . 2011 Role of magnesium in alleviation of aluminium toxicity in plants. J. Exp. Bot. 62:2251–2264. 21273333 10.1093/jxb/erq456

[ece31835-bib-0004] Brunner, I. , and C. Sperisen . 2013 Aluminum exclusion and aluminum tolerance in woody plants. Front. Plant Sci. 4:1–12. 23781222 10.3389/fpls.2013.00172 PMC3679494

[ece31835-bib-0005] Burslem, D. F. R. P. , P. J. Grubb , and I. M. Turner . 1995 Responses to nutrient addition among shade‐tolerant tree species of lowland rain‐forest in Singapore. J. Ecol. 83:113–122.

[ece31835-bib-0006] Burslem, D. F. R. P. , P. J. Grubb , and I. M. Turner . 1996 Responses to simulated drought and elevated nutrient supply among shade‐tolerant tree seedlings of lowland tropical forest in Singapore. Biotropica 28:636–648.

[ece31835-bib-0007] Cakmak, I. , and E. A. Kirkby . 2008 Role of magnesium in carbon partitioning and alleviating photooxidative damage. Physiol. Plant. 133:692–704. 18724409 10.1111/j.1399-3054.2007.01042.x

[ece31835-bib-0008] Cakmak, I. , C. Hengeler , and H. Marschner . 1994a Changes in phloem export of sucrose in leaves in response to phosphorus, potassium and magnesium deficiency in bean plants. J. Exp. Bot. 45:1251–1257.

[ece31835-bib-0009] Cakmak, I. , C. Hengeler , and H. Marschner . 1994b Partitioning of shoot and root dry matter and carbohydrates in bean plants suffering from phosphorus, potassium and magnesium deficiency. J. Exp. Bot. 45:1245–1250.

[ece31835-bib-0010] Chuyong, C. B. , D. M. Newbery , and N. C. Songwe . 2000 Litter nutrients and retranslocation in a central African rain forest dominated by ectomycorrhizal trees. New Phytol. 148:493–510. 10.1046/j.1469-8137.2000.00774.x 33863026

[ece31835-bib-0011] Chuyong, G. B. , D. M. Newbery , and N. C. Songwe . 2002 Litter breakdown and mineralization in a central African rain forest dominated by ectomycorrhizal trees. Biogeochemistry 61:73–94. 10.1046/j.1469-8137.2000.00774.x 33863026

[ece31835-bib-0012] Chuyong, G. B. , D. M. Newbery , and N. C. Songwe . 2004 Rainfall input, throughfall and stemflow of nutrients in a central African rain forest dominated by ectomycorrhizal trees. Biogeochemistry 67:73–91. 10.1046/j.1469-8137.2000.00774.x 33863026

[ece31835-bib-0013] Ding, Y. , W. Luo , and G. Xu . 2006 Characterisation of magnesium nutrition and interaction of magnesium and potassium in rice. Ann. Appl. Biol. 149:111–123.

[ece31835-bib-0014] Epstein, E. , and A. J. Bloom . 2005 Mineral nutrition of plants: principles and perspectives. Sinauer Associates, Sunderland, MA.

[ece31835-bib-0015] Ericsson, T. , and M. Kahr . 1995 Growth and nutrition of birch seedlings at varied relative addition rates of magnesium. Tree Physiol. 15:85–93. 14965980 10.1093/treephys/15.2.85

[ece31835-bib-0016] Gartlan, J. S. 1992 Cameroon Pp. 110–118 *in* Sayer J. A., Harcourt C. S. and Collins N. M., eds. The conservation atlas of tropical forests: Africa. Macmillan Publishers, London, U.K.

[ece31835-bib-0017] Gartlan, J. S. , D. M. Newbery , D. W. Thomas , and P. G. Waterman . 1986 The influence of topography and soil phosphorus on the vegetation of Korup Forest Reserve, Cameroun. Vegetatio 65:131–148.

[ece31835-bib-0018] Green, J. J. , and D. M. Newbery . 2001a Light and seed size affect establishment of grove‐forming ectomycorrhizal rain forest tree species. New Phytol. 151:271–289. 10.1046/j.1469-8137.2001.00156.x 33873393

[ece31835-bib-0019] Green, J. J. , and D. M. Newbery . 2001b Shade and leaf loss affect establishment of grove‐forming ectomycorrhizal rain forest tree species. New Phytol. 151:291–309. 10.1046/j.1469-8137.2001.00157.x 33873392

[ece31835-bib-0020] Green, J. J. , and D. M. Newbery . 2002 Reproductive investment and seedling survival of the mast‐fruiting rain forest tree, *Microberlinia bisulcata* A. Chev. Plant Ecol. 162:169–183.

[ece31835-bib-0021] Gunatilleke, C. V. S. , I. Gunatilleke , G. A. D. Perera , D. Burslem , P. M. S. Ashton , and P. S. Ashton . 1997 Responses to nutrient addition among seedlings of eight closely related species of Shorea in Sri Lanka. J. Ecol. 85:301–311.

[ece31835-bib-0022] Hawkins, P. , and M. Brunt . 1965 The soil and ecology of West Cameroon. Report to Government of Cameroun. No. 2083. Vol. I, FAO, Rome.

[ece31835-bib-0023] Hermans, C. , G. N. Johnson , R. J. Strasser , and N. Verbruggen . 2004 Physiological characterisation of magnesium deficiency in sugar beet: acclimation to low magnesium differentially affects photosystems I and II. Planta 220:344–355. 15378366 10.1007/s00425-004-1340-4

[ece31835-bib-0024] Hermans, C. , F. Bourgis , M. Faucher , R. J. Strasser , S. Delrot , and N. Verbruggen . 2005 Magnesium deficiency in sugar beets alters sugar partitioning and phloem loading in young mature leaves. Planta 220:541–549. 15580527 10.1007/s00425-004-1376-5

[ece31835-bib-0025] Hermans, C. , J. P. Hammond , P. J. White , and N. Verbruggen . 2006 How do plants respond to nutrient shortage by biomass allocation? Trends Plant Sci. 11:610–617. 17092760 10.1016/j.tplants.2006.10.007

[ece31835-bib-0026] Holste, E. K. , R. K. Kobe , and C. F. Vriesendorp . 2011 Seedling growth responses to soil resources in the understory of a wet tropical forest. Ecology 92:1828–1838. 21939079 10.1890/10-1697.1

[ece31835-bib-0027] Jentschke, G. , B. Brandes , A. J. Kuhn , W. H. Schroder , J. S. Becker , and D. L. Godbold . 2000 The mycorrhizal fungus *Paxillus involutus* transports magnesium to Norway spruce seedlings. Evidence from stable isotope labeling. Plant Soil 220:243–246.

[ece31835-bib-0028] Jentschke, G. , B. Brandes , A. J. Kuhn , W. H. Schroder , and D. L. Godbold . 2001 Interdependence of phosphorus, nitrogen, potassium and magnesium translocation by the ectomycorrhizal fungus *Paxillus involutus* . New Phytol. 149:327–337. 10.1046/j.1469-8137.2001.00014.x 33874636

[ece31835-bib-0029] Juliana, W. A. W. , D. F. R. P. Burslem , and M. D. Swaine . 2009 Nutrient limitation of seedling growth on contrasting soils from Pasoh Forest Reserve, Peninsular Malaysia. J. Trop. For. Sci. 21:316–327.

[ece31835-bib-0030] Laing, W. , D. Greer , O. Sun , P. Beets , A. Lowe , and T. Payn . 2000 Physiological impacts of Mg deficiency in *Pinus radiata*: growth and photosynthesis. New Phytol. 146:47–57.

[ece31835-bib-0031] Letouzey, R. 1968 Etude phytogéographique du Cameroun. P. Le Chevalier, Paris.

[ece31835-bib-0032] Letouzey, R. 1985 Notice de la carte phytogéographique du Cameroun au 1:500,000. Institut de la Carte Internationale de la Végétation, Toulouse.

[ece31835-bib-0033] Marschner, H. 1995 Mineral nutrition of higher plants, 2nd ed. Academic Press, London.

[ece31835-bib-0034] McDonald, A. J. S. , T. Ericsson , and C. M. Larsson . 1996 Plant nutrition, dry matter gain and partitioning at the whole‐plant level. J. Exp. Bot. 47:1245–1253. 21245256 10.1093/jxb/47.Special_Issue.1245

[ece31835-bib-0035] Mehne‐Jakobs, B. 1995 The influence of magnesium deficiency on carbohydrate concentrations in Norway spruce (*Picea abies*) needles. Tree Physiol. 15:577–584. 14965915 10.1093/treephys/15.9.577

[ece31835-bib-0036] Mehne‐Jakobs, B. . 1996 Magnesium deficiency treatment causes reductions in photosynthesis of well‐nourished Norway spruce. Trees‐Struct. Funct. 10:293–300.

[ece31835-bib-0037] Mengel, K. 2007 Potassium Pp. 91–120 *in* Barker A. V. and Pilbeam D. J., eds. Handbook of plant nutrition. CRC/Taylor & Francis, Boca Raton.

[ece31835-bib-0038] Merhaut, D. J. 2007 Magnesium Pp. 145–181 *in* Barker A. V., Pilbeam D. J., eds. Handbook of plant nutrition. CRC/Taylor & Francis, Boca Raton.

[ece31835-bib-0039] Mitchell, A. D. , P. Loganathan , T. W. Payn , and R. W. Tillman . 1999 Effect of calcined magnesite on soil and *Pinus radiata* foliage magnesium in pumice soils of New Zealand. Aust. J. Soil Res. 37:545–560.

[ece31835-bib-0040] Miyasaka, S. C. , N. V. Hue , and M. A. Dunn . 2007 Aluminum Pp. 439–497 *in* Barker A. V. and Pilbeam D. J., eds. Handbook of plant nutrition. CRC/Taylor & Francis, Boca Raton.

[ece31835-bib-0041] Newbery, D. M. , and J. S. Gartlan . 1996 Structural analysis of the rain forest at Korup and Douala Edea, Cameroon. Proc. R. Soc. Edinb. B 104:177–224.

[ece31835-bib-0042] Newbery, D. M. , I. J. Alexander , D. W. Thomas , and J. S. Gartlan . 1988 Ectomycorrhizal rain‐forest legumes and soil phosphorus in Korup‐National‐Park, Cameroon. New Phytol. 109:433–450.

[ece31835-bib-0043] Newbery, D. M. , I. J. Alexander , and J. A. Rother . 1997 Phosphorus dynamics in a lowland African rain forest: the influence of ectomycorrhizal trees. Ecol. Monogr. 67:367–409.

[ece31835-bib-0044] Newbery, D. M. , N. C. Songwe , and G. B. Chuyong . 1998 Phenology and dynamics of an African rain forest at Korup, Cameroon Pp. 267–308 *in* Newbery D. M., Prins H. H. T. and Brown N. D., eds. Dynamics of tropical communities. Blackwell Science, Oxford.

[ece31835-bib-0045] Newbery, D. M. , I. J. Alexander , and J. A. Rother . 2000 Does proximity to conspecific adults influence the establishment of ectomycorrhizal trees in rain forest? New Phytol. 147:401–409.

[ece31835-bib-0046] Newbery, D. M. , G. B. Chuyong , J. J. Green , N. C. Songwe , F. Tchuenteu , and L. Zimmermann . 2002 Does low phosphorus supply limit seedling establishment and tree growth in groves of ectomycorrhizal trees in a central African rainforest? New Phytol. 156:297–311. 10.1046/j.1469-8137.2002.00505.x 33873273

[ece31835-bib-0047] Newbery, D. M. , X. M. van der Burgt , and M. A. Moravie . 2004 Structure and inferred dynamics of a large grove of *Microberlinia bisulcata* trees in central African rain forest: the possible role of periods of multiple disturbance events. J. Trop. Ecol. 20:131–143.

[ece31835-bib-0048] Newbery, D. M. , G. B. Chuyong , and L. Zimmermann . 2006a Mast fruiting of large ectomycorrhizal African rain forest trees: importance of dry season intensity, and the resource‐limitation hypothesis. New Phytol. 170:561–579. 16626477 10.1111/j.1469-8137.2006.01691.x

[ece31835-bib-0049] Newbery, D. M. , G. B. Chuyong , L. Zimmermann , and C. Praz . 2006b Seedling survival and growth of three ectomycorrhizal caesalpiniaceous tree species in a Central African rain forest. J. Trop. Ecol. 22:499–511.

[ece31835-bib-0050] Newbery, D. M. , S. Schwan , G. B. Chuyong , and X. M. van der Burgt . 2009 Buttress form of the central African rain forest tree *Microberlinia bisulcata*, and its possible role in nutrient acquisition. Trees‐Struct. Funct. 23:219–234.

[ece31835-bib-0051] Newbery, D. M. , C. J. Praz , X. M. van der Burgt , J. M. Norghauer , and G. B. Chuyong . 2010 Recruitment dynamics of the grove‐dominant tree *Microberlinia bisulcata* in African rain forest: extending the light response versus adult longevity trade‐off concept. Plant Ecol. 206:151–172.

[ece31835-bib-0052] Newbery, D. M. , X. M. van der Burgt , M. Worbes , and G. B. Chuyong . 2013 Transient dominance in a central African rain forest. Ecol. Monogr. 83:339–382.

[ece31835-bib-0053] Nilus, R. , C. R. Maycock , N. Majalap‐Lee , and D. F. R. P. Burslem . 2011 Nutrient limitation of tree seedling growth in three soil types found in Sabah. J. Trop. For. Sci. 23:133–142.

[ece31835-bib-0054] Norghauer, J. M. , and D. M. Newbery . 2010 Recruitment limitation after mast‐seeding in two African rain forest trees. Ecology 91:2303–2312. 20836452 10.1890/09-0071.1

[ece31835-bib-0055] Norghauer, J. M. , and D. M. Newbery . 2011 Seed fate and seedling dynamics after masting in two African rain forest trees. Ecol. Monogr. 81:443–468.

[ece31835-bib-0056] Norghauer, J. M. , and D. M. Newbery . 2013 Herbivores equalize the seedling height growth of three dominant tree species in an African tropical rain forest. For. Ecol. Manage. 310:555–566.

[ece31835-bib-0057] Norghauer, J. M. , and D. M. Newbery . 2014 Herbivores differentially limit the seedling growth and sapling recruitment of two dominant rain forest trees. Oecologia 174:459–469. 24072438 10.1007/s00442-013-2769-6

[ece31835-bib-0058] Norghauer, J. M. , and D. M. Newbery . 2015 Tree size and fecundity influence ballistic seed dispersal of two dominant mast‐fruiting species in a tropical rain forest. For. Ecol. Manage. 338:100–113.

[ece31835-bib-0059] Norghauer, J. M. , D. M. Newbery , L. Tedersoo , and G. B. Chuyong . 2010 Do fungal pathogens drive density‐dependent mortality in established seedlings of two dominant African rain‐forest trees? J. Trop. Ecol. 26:293–301.

[ece31835-bib-0060] Norghauer, J. M. , G. Glauser , and D. M. Newbery . 2014 Seedling resistance, tolerance and escape from herbivores: insights from co‐dominant canopy tree species in a resource‐poor African rain forest. Funct. Ecol. 28:1426–1439.

[ece31835-bib-0061] Payne, R. W. , S. A. Harding , D. A. Murray , D. M. Soutar , D. B. Baird , A. I. Glaser , et al. 2011 The guide to GenStat release 14, Part 1: Syntax and data management. Part 2: statistics. VSN International, Hemel Hempstead, U.K.

[ece31835-bib-0062] Rubio, G. , J. M. Zhu , and J. P. Lynch . 2003 A critical test of the two prevailing theories of plant response to nutrient availability. Am. J. Bot. 90:143–152. 21659090 10.3732/ajb.90.1.143

[ece31835-bib-0063] Santiago, L. S. , S. J. Wright , K. E. Harms , J. B. Yavitt , C. Korine , M. N. Garcia , et al. 2012 Tropical tree seedling growth responses to nitrogen, phosphorus and potassium addition. J. Ecol. 100:309–316.

[ece31835-bib-0064] Schwan, S. 2003 Phenology, resource conservation and tree architecture of large ectomycorrhizal trees in a lowland African rainforest at Korup, Cameroon. Unpublished MSc, thesis. University of Bern: 75 pp.

[ece31835-bib-0065] Sun, O. J. , and T. W. Payn . 1999 Magnesium nutrition and photosynthesis in *Pinus radiata*: clonal variation and influence of potassium. Tree Physiol. 19:535–540. 12651544 10.1093/treephys/19.8.535

[ece31835-bib-0066] Tahara, K. , M. Norisada , T. Tange , H. Yagi , and K. Kojima . 2005 Ectomycorrhizal association enhances Al tolerance by inducing citrate secretion in *Pinus densiflora* . Soil Sci. Plant Nutr. 51:397–403.

[ece31835-bib-0067] Turner, I. M. , N. D. Brown , and A. C. Newton . 1993 The effect of fertilizer application on dipterocarp seedling growth and mycorrhizal infection. For. Ecol. Manage. 57:329–337.

[ece31835-bib-0068] Wright, S. J. , J. B. Yavitt , N. Wurzburger , B. L. Turner , E. V. J. Tanner , E. J. Sayer , et al. 2011 Potassium, phosphorus, or nitrogen limit root allocation, tree growth, or litter production in a lowland tropical forest. Ecology 92:1616–1625. 21905428 10.1890/10-1558.1

[ece31835-bib-0069] Yang, J. L. , J. F. You , Y. Y. Li , P. Wu , and S. J. Zheng . 2007 Magnesium enhances aluminum‐induced citrate secretion in rice bean roots (*Vigna umbellata*) by restoring plasma membrane H + ‐ATPase activity. Plant Cell Physiol. 48:66–73. 17132634 10.1093/pcp/pcl038

[ece31835-bib-0070] Yang, G. H. , L. T. Yang , H. X. Jiang , Y. Li , P. Wang , and L. S. Chen . 2012 Physiological impacts of magnesium‐deficiency in *Citrus* seedlings: photosynthesis, antioxidant system and carbohydrates. Trees‐Struct. Funct. 26:1237–1250.

