# Corrigendum

**DOI:** 10.1002/ece3.7356

**Published:** 2021-05-03

In “Limitation of seedling growth by potassium and magnesium supply for two ectomycorrhizal tree species of a Central African rain forest and its implication for their recruitment”, which was published in volume 6 issue 1, January 2016, on page 127, fourth paragraph of the Methods section it was stated:

The proportion of K_2_O in potash was 50.11%, and accordingly the levels of K applied were: (1) no fertilizer; (2) 5 g potash > 2.08 g K (low); (3) 10 g potash > 4.16 g K (intermediate); and (4) 15 g potash > = 6.24 g K (high). The proportion of MgO in kieserite was 27.65%, and likewise the levels of Mg applied were: (1) no fertilizer; (2) 5 g kieserite > 0.83 g Mg (low); (3) 10 g kieserite > 1.68 g Mg (intermediate); and (4) 15 g kieserite > 2.49 g Mg (high).

The correct statement should be:

The proportion of K_2_O‐equivalent in potash was 50.11%, and accordingly, the levels of K applied were: (1) no fertilizer; (2) 5 g potash > 2.08 g K (low); (3) 10 g potash > 4.16 g K (intermediate); and (4) 15 g potash > = 6.24 g K (high). The proportion of MgO‐equivalent in kieserite was 27.65%, and likewise the levels of Mg applied were: (1) no fertilizer; (2) 5 g kieserite > 0.83 g Mg (low); (3) 10 g kieserite > 1.68 g Mg (intermediate); and (4) 15 g kieserite > 2.49 g Mg (high).

The results and conclusions of the article are unaffected by these amendments.
