# Supplementary figures and images for: Limitation of seedling growth by potassium and magnesium supply for two ectomycorrhizal tree species of a Central African rain forest and its implication for their recruitment
Source: Ecol Evol. 2015 Dec 15;6(1):125–42. doi: 10.1002/ece3.1835 (PMC4716515; doi:10.1002/ece3.1835)

*Microberlinia bisulcata* (upper), *Tetraberlinia bifoliolata* (lower).

Fig. S1

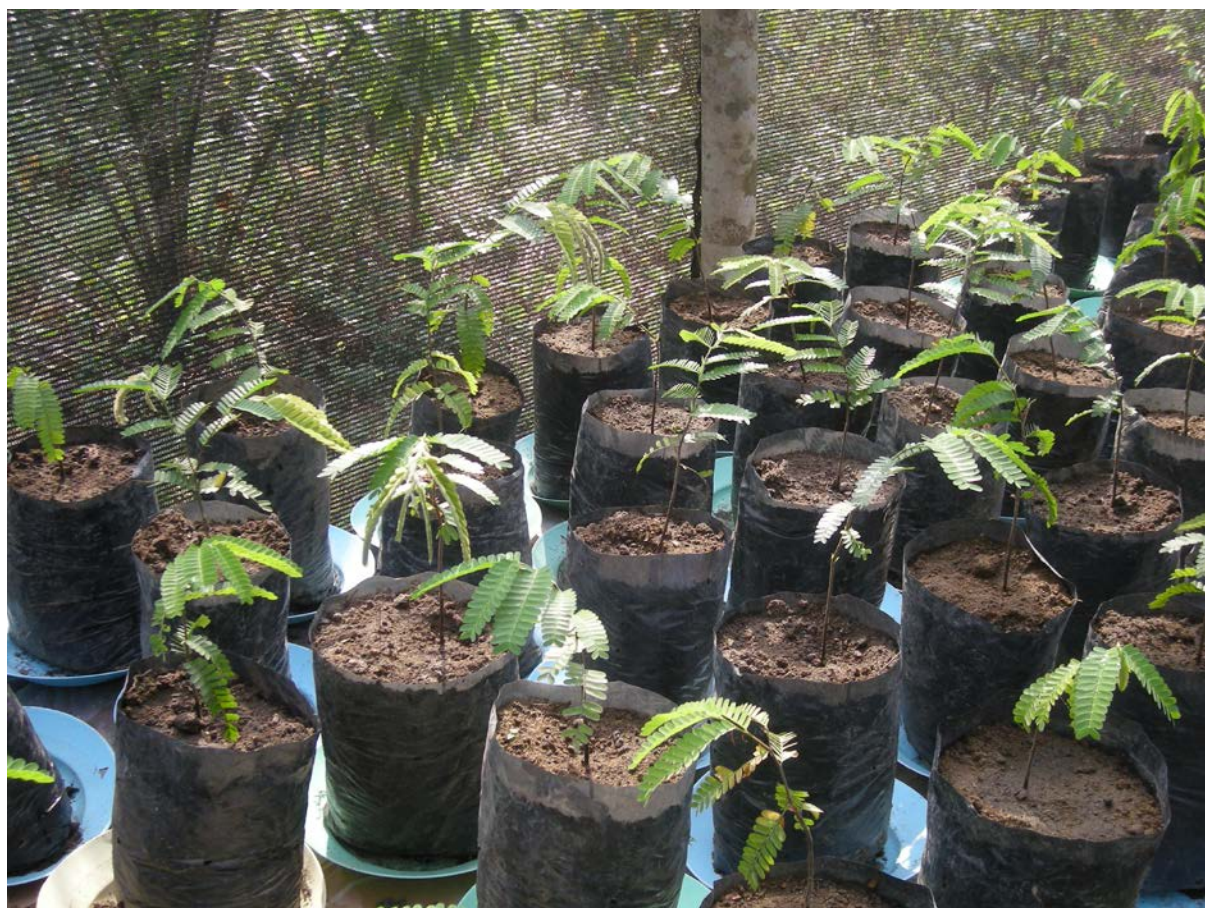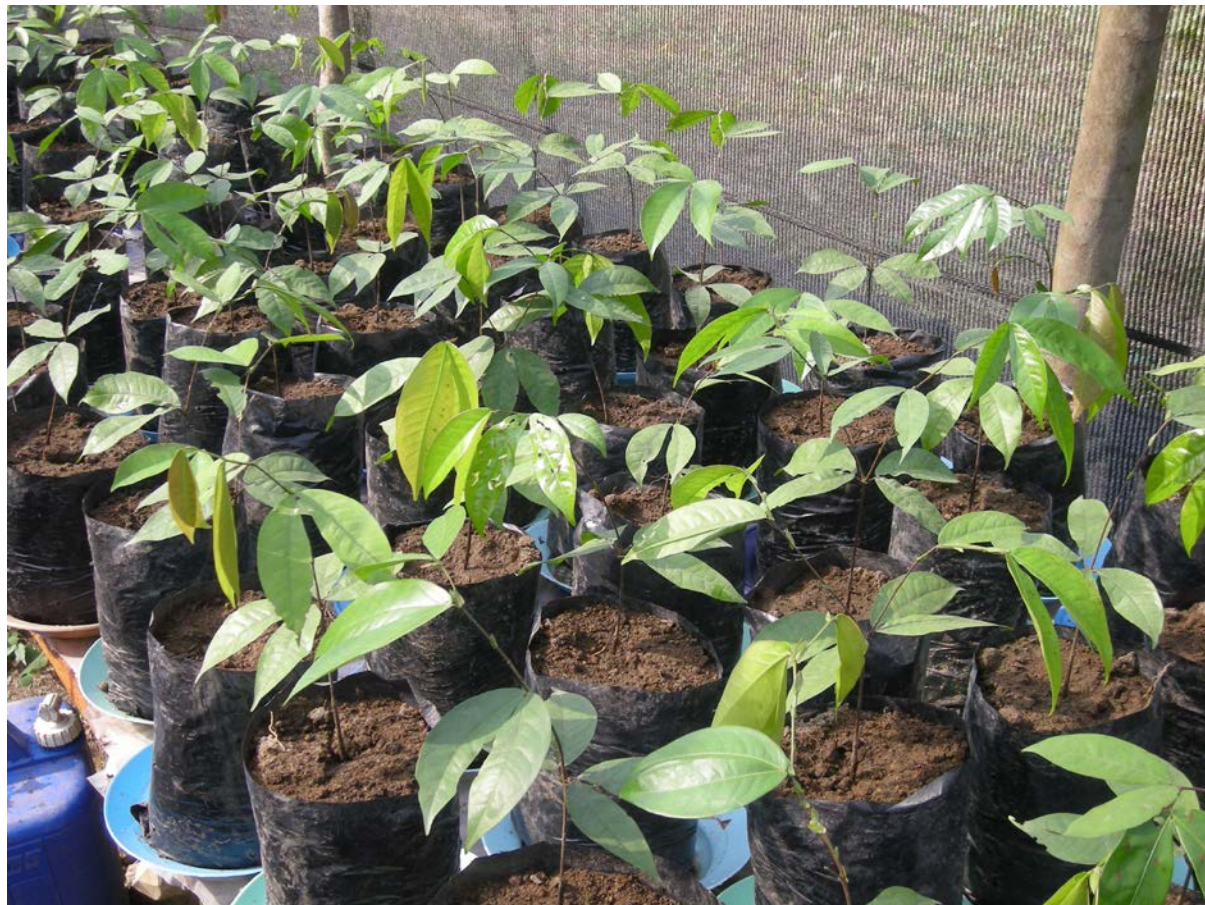

Supplement: Supplementary file 1 — Figure S1. Microberlinia bisulcata (Mb) and Tetraberlinia bifoliolata (Tb) growing within the shade house at the Mana Bridge Nursery, next to Korup, Ndian. [file ECE3-6-125-s001.pdf]

**root**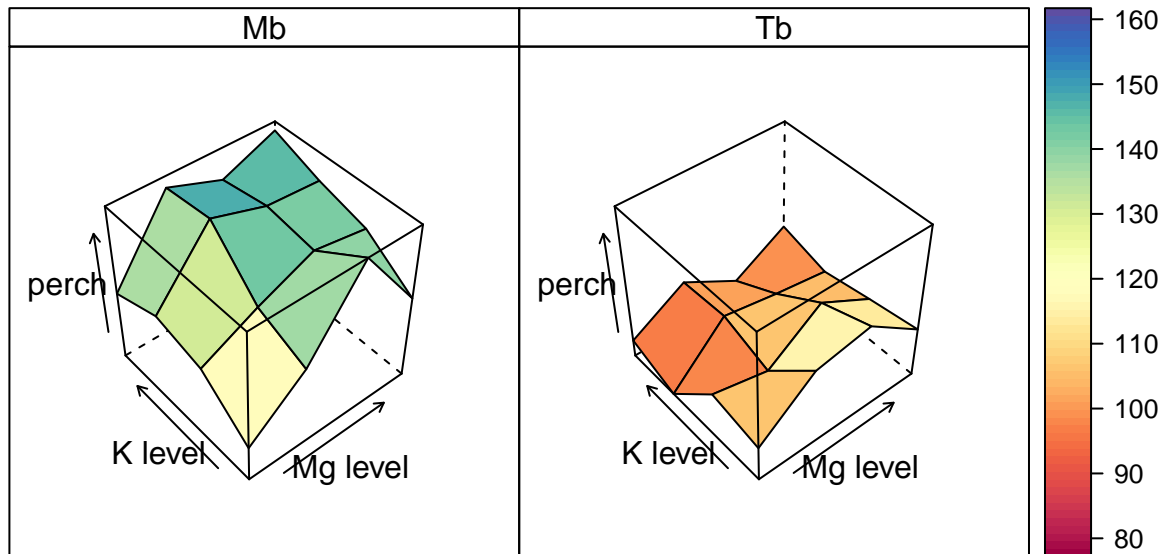**stem**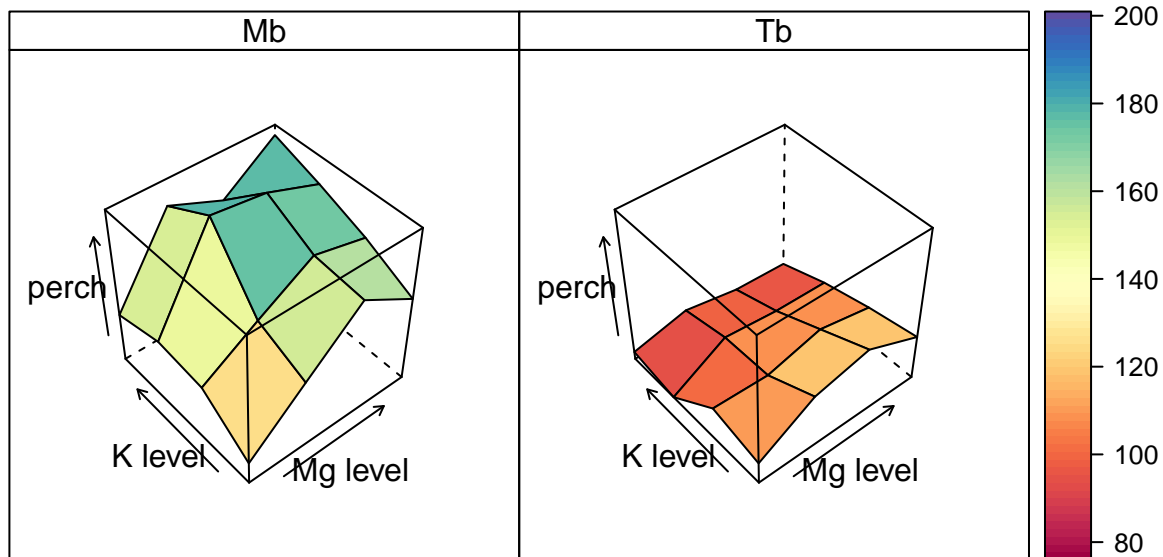

Supplement: Supplementary file 2 — Figure S2. Percentage change (perch) in dry mass of the two species Microberlinia bisulcata (Mb) and Tetraberlinia bifoliolata (Tb) for the 16 treatments (four levels of K x four levels for Mg; each factor being null and three increasing additions), averaged across the three harvests, with reference to the common control [1,1] for (a) root, and (b) shoot parts. This complements leaf and total parts in Fig. 2. [file ECE3-6-125-s002.pdf]

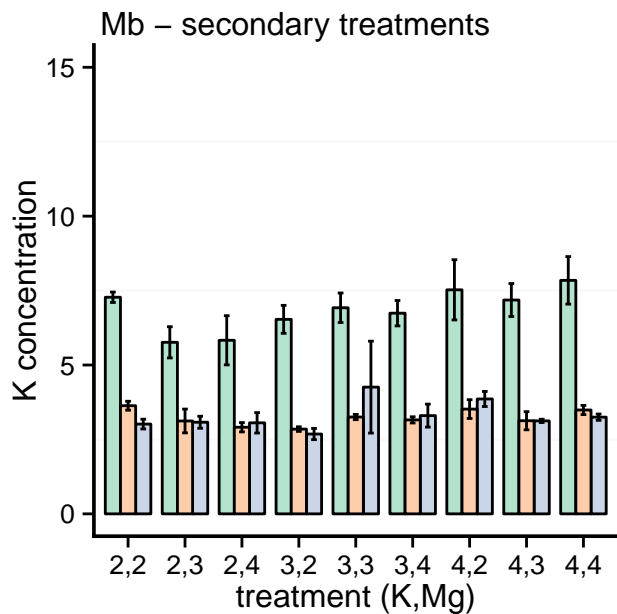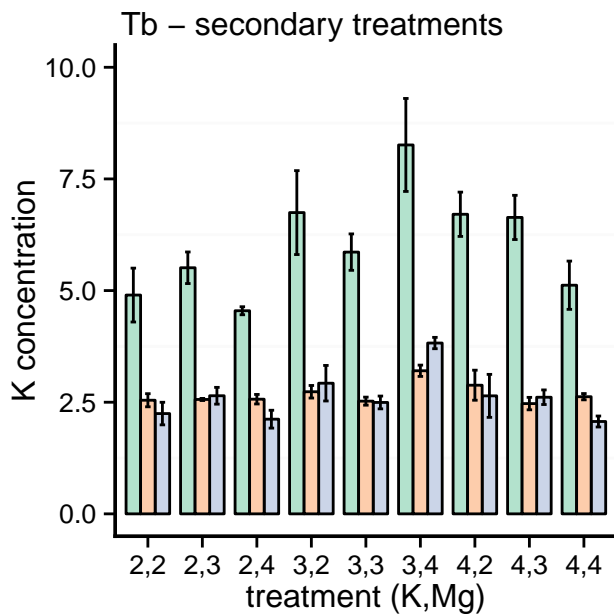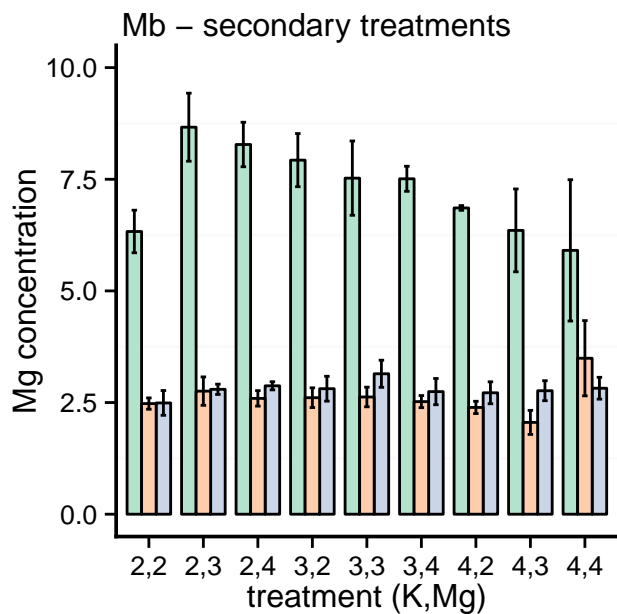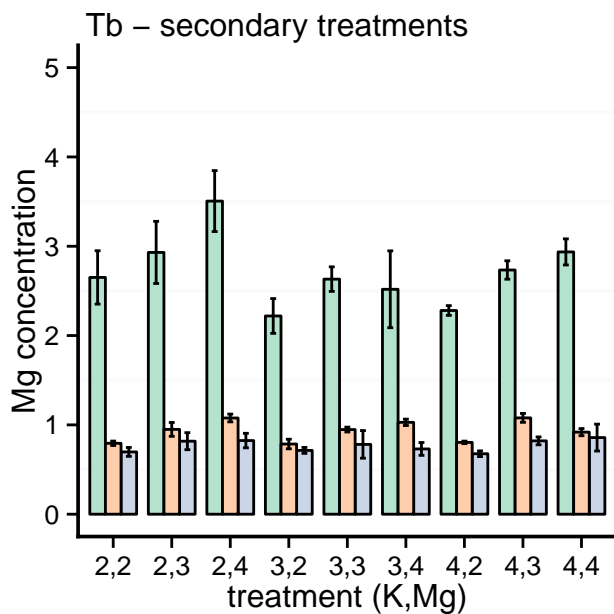

Supplement: Supplementary file 3 — Figure S3. Mean concentrations (mg/g) of potassium and magnesium, in leaves (green), roots (brown) and stems (light blue bars) of seedlings of Microberlinia bisulcata (Mb) and Tetraberlinia bifoliolata (Tb) at harvest 2 for the secondary treatments. A secondary treatment had no factor at level 1. Concentrations for corresponding primary treatments are shown in Fig. 3. [file ECE3-6-125-s003.pdf]

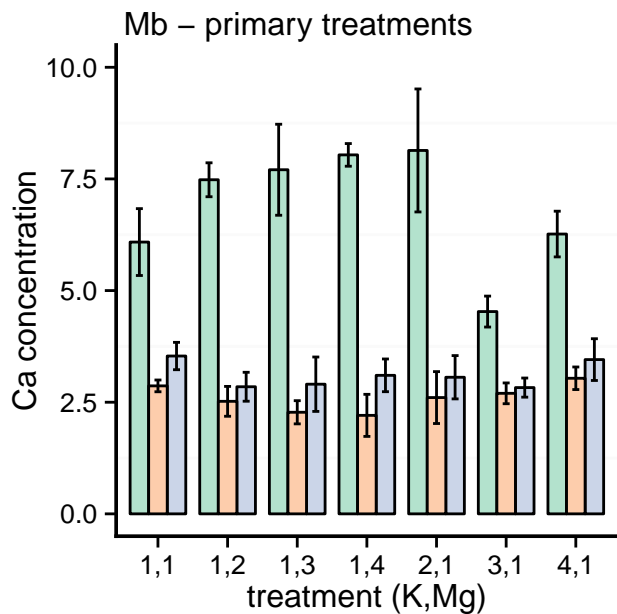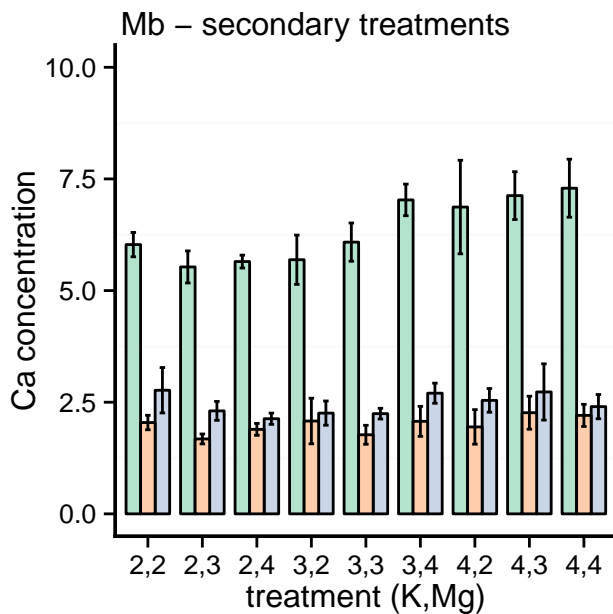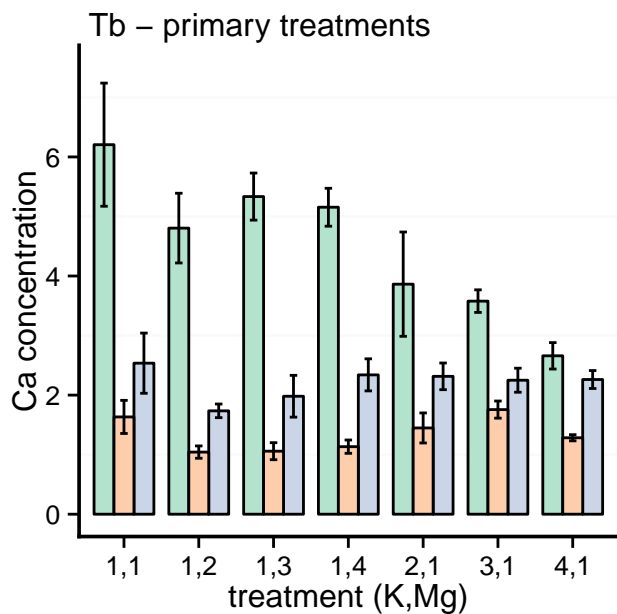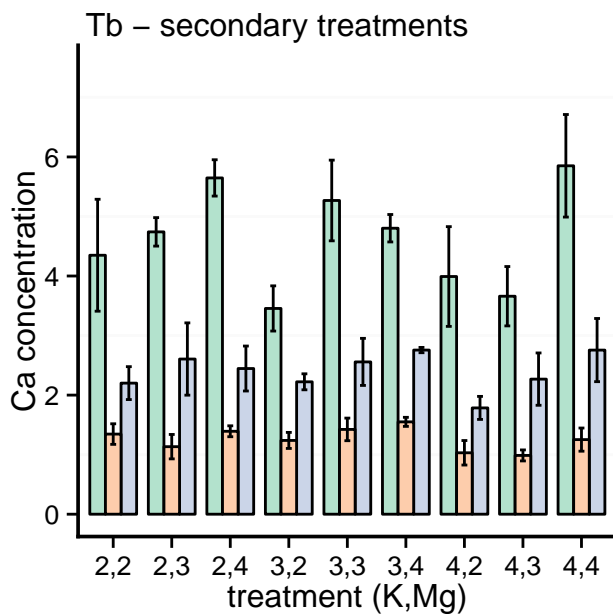

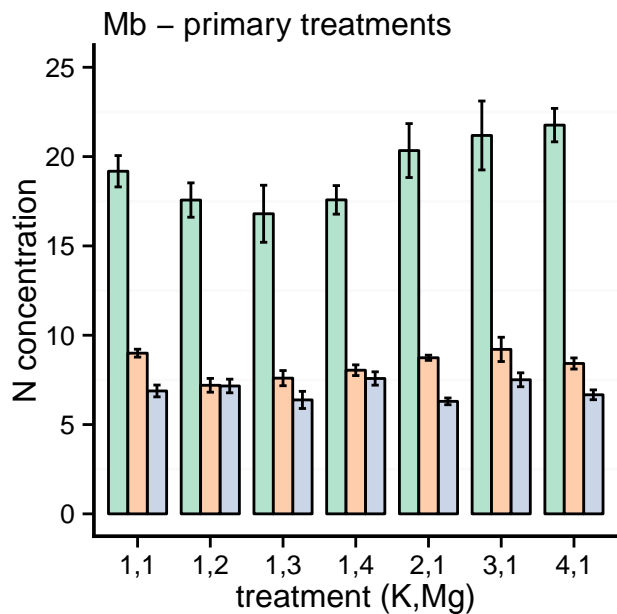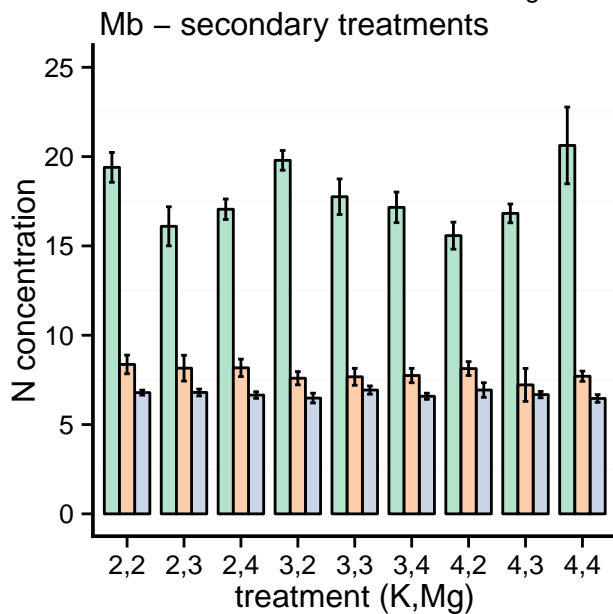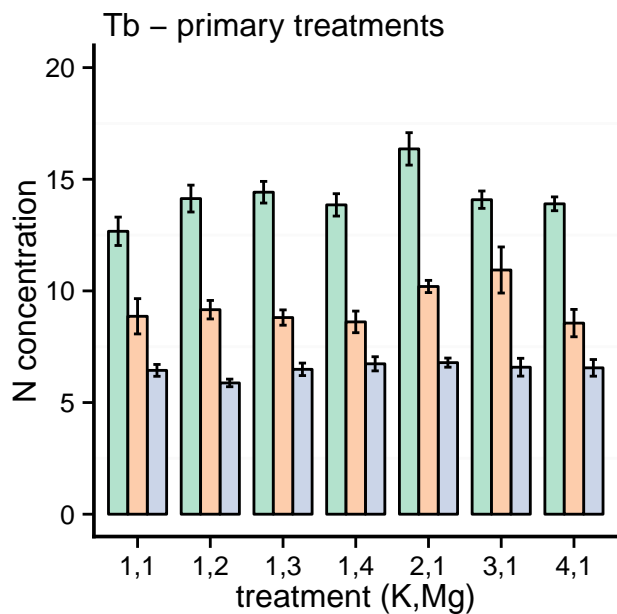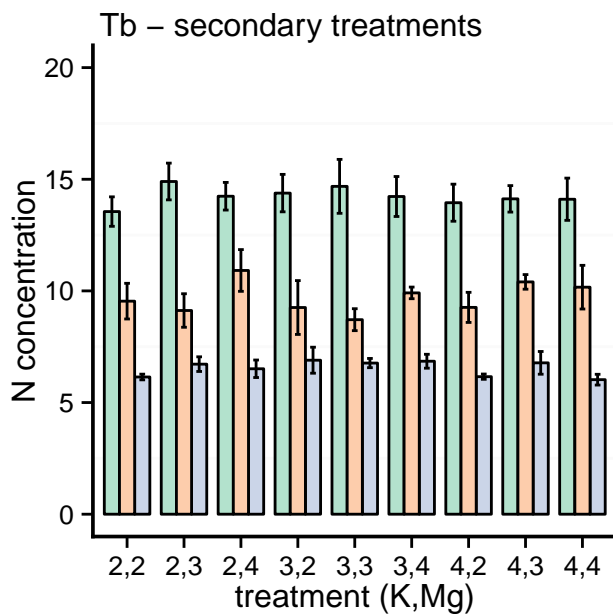

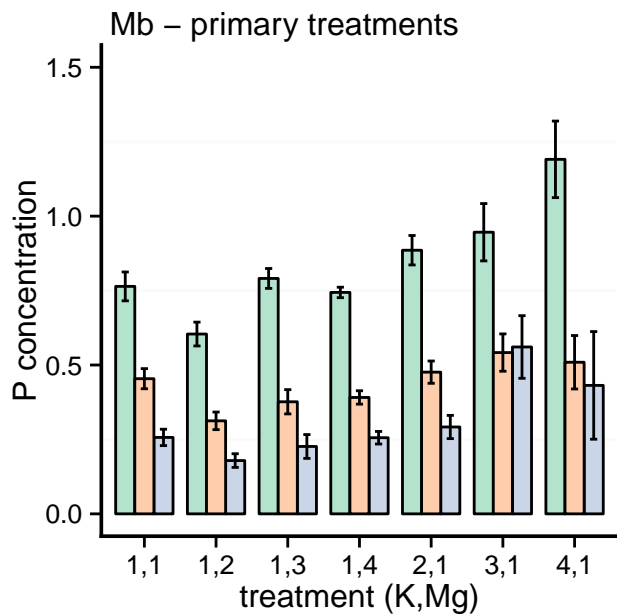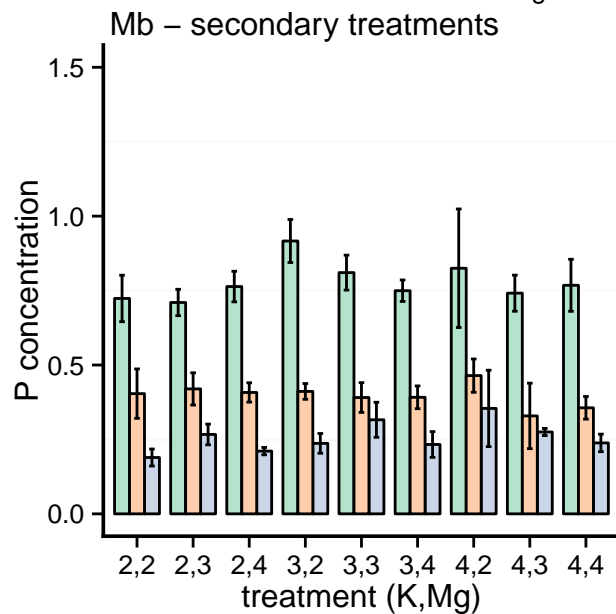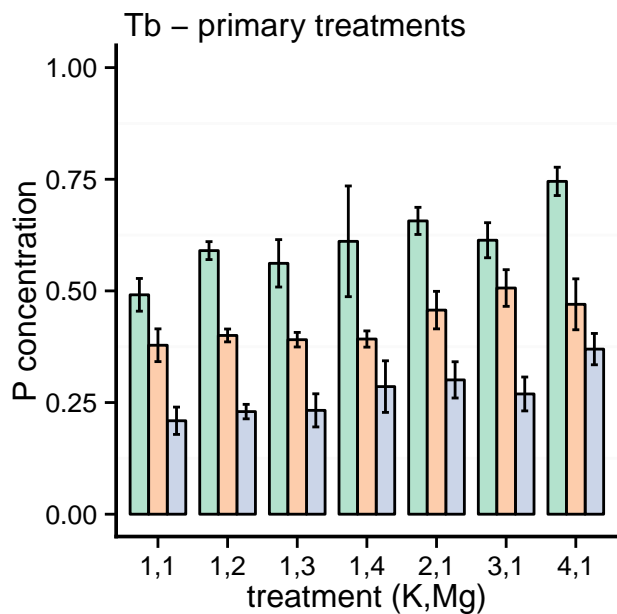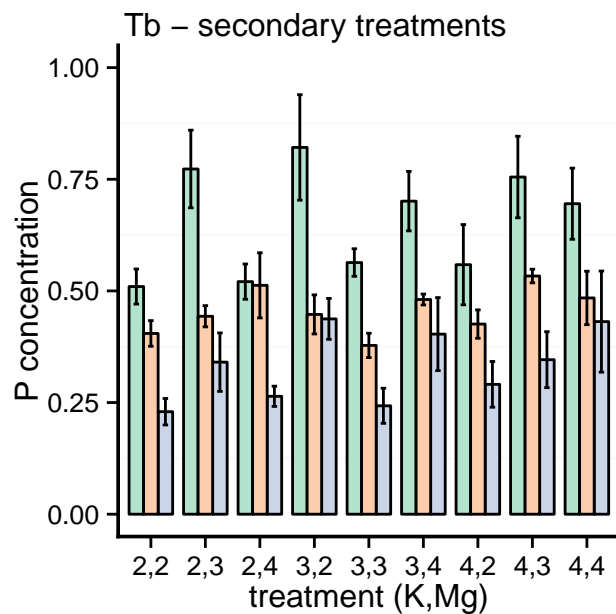

Supplement: Supplementary file 4 — Figure S4. Mean concentrations (mg/g) of (a) calcium, (b) nitrogen, and (c) phosphorus, in leaves (green), roots (brown) and stems (light blue bars) of seedlings of Microberlinia bisulcata (Mb) and Tetraberlinia bifoliolata (Tb) at harvest 2 for the primary and secondary treatments. A primary treatment had at least one factor at level 1, whilst secondary treatments had none. [file ECE3-6-125-s004.pdf]

**Ca**

Fig. S5

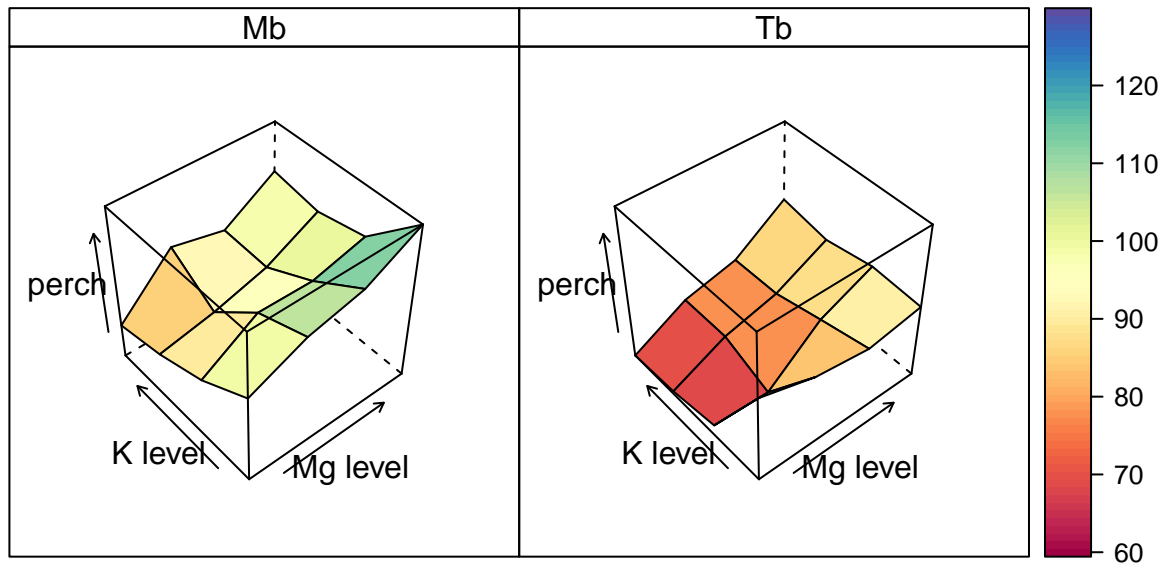**N**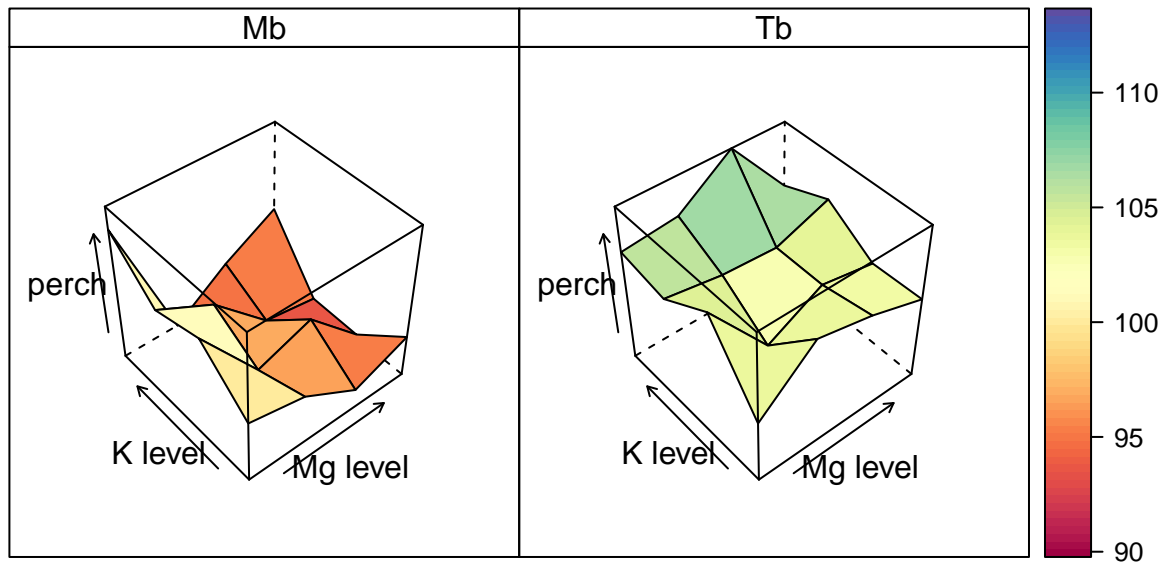

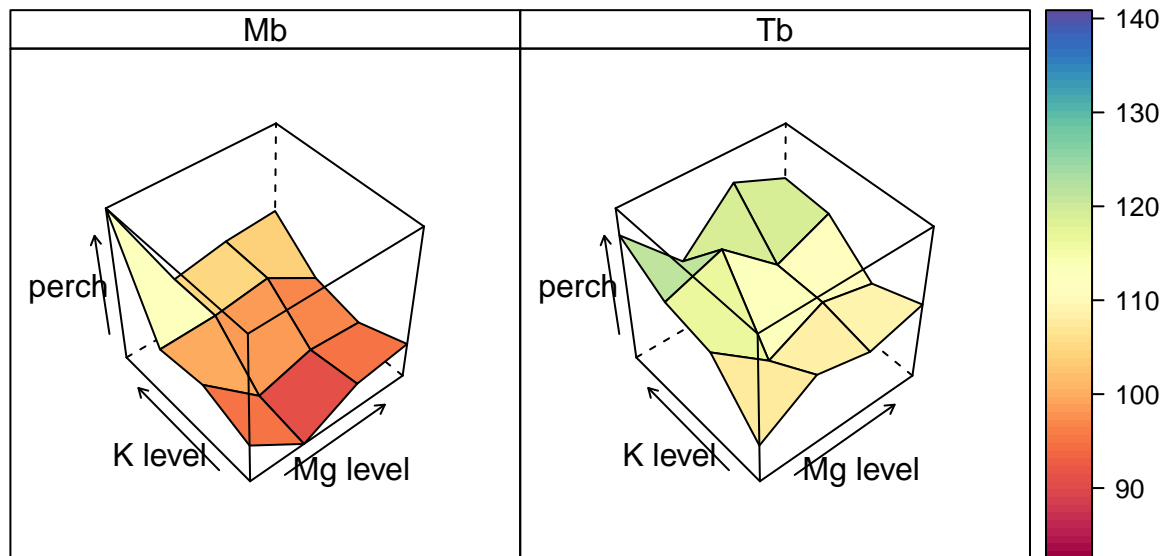

Supplement: Supplementary file 5 — Figure S5. Percentage change (perch) in calcium, nitrogen and phosphorus leaf nutrient concentrations of Microberlinia bisulcata (Mb) and Tetraberlinia bifoliolata (Tb) for the 16 treatments with reference to the common control. Explanations of factor levels and perch are given in Fig. 2. [file ECE3-6-125-s005.pdf]

## K – upper

Fig. S6a

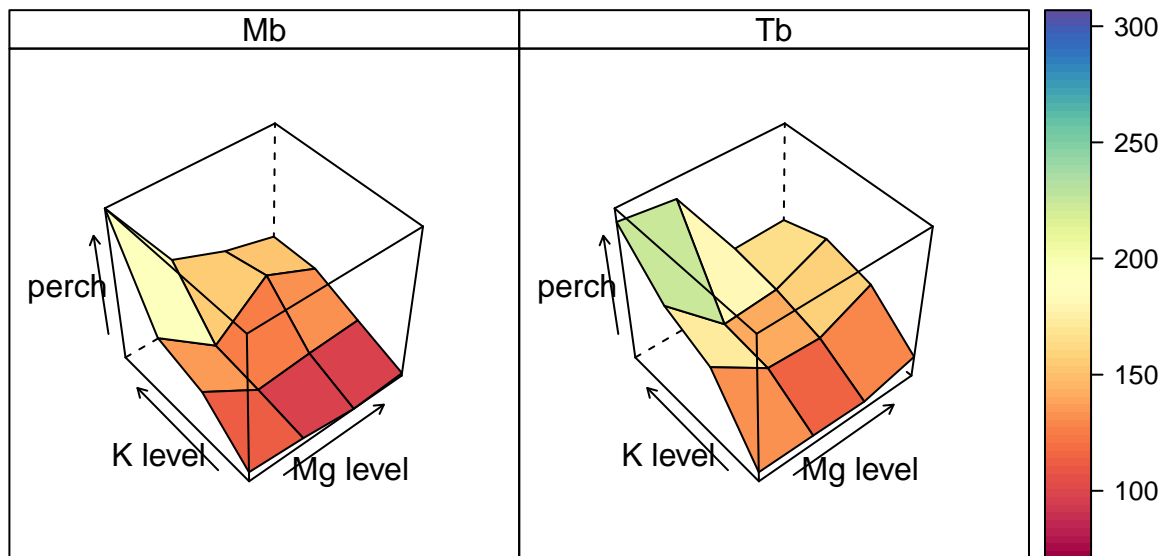

## K – lower

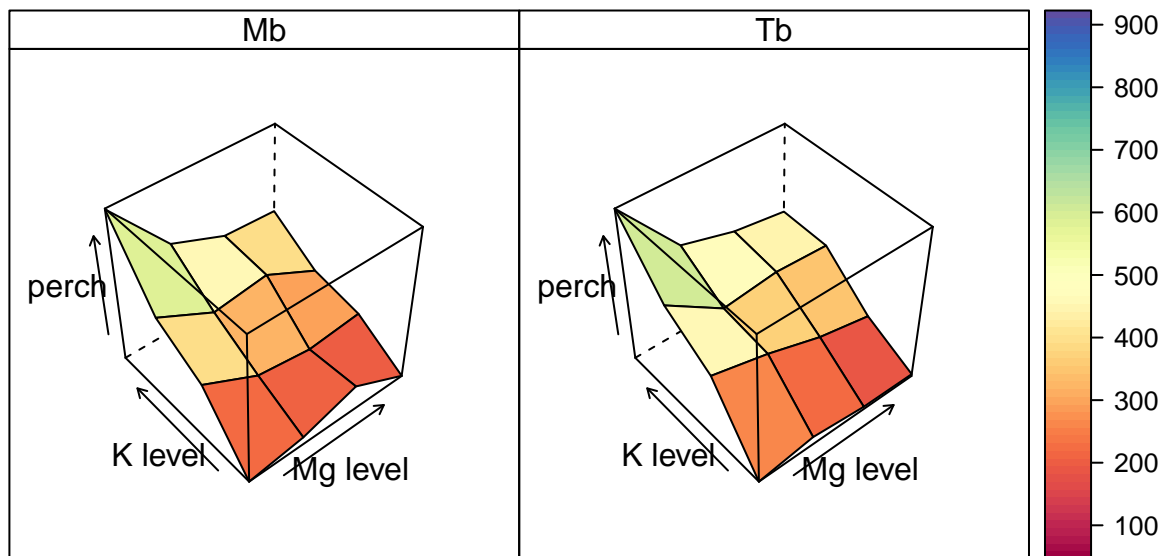

## Mg – upper

Fig. S6b

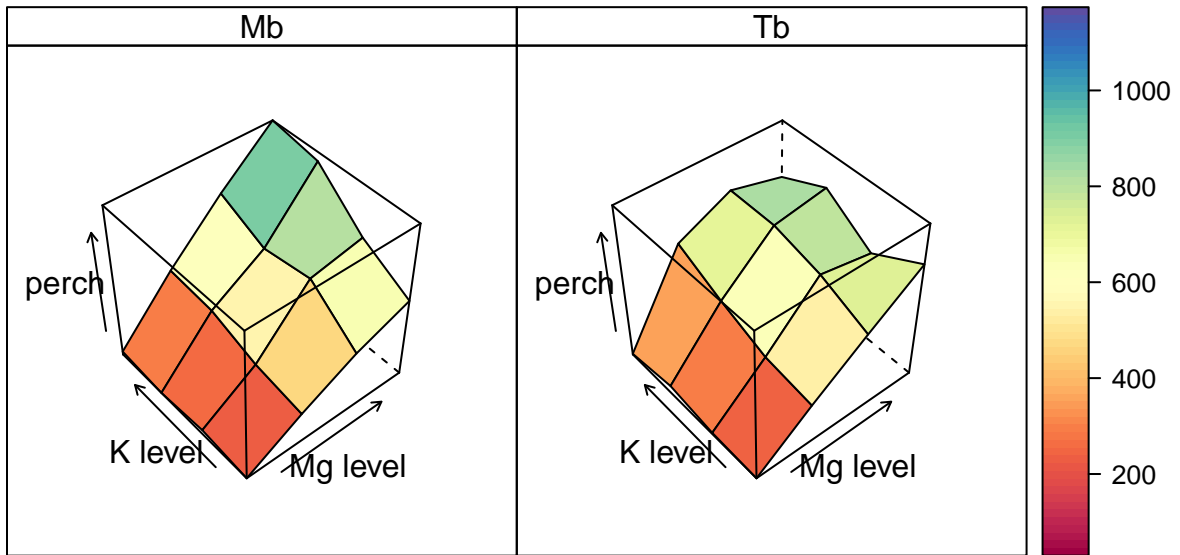

## Mg – lower

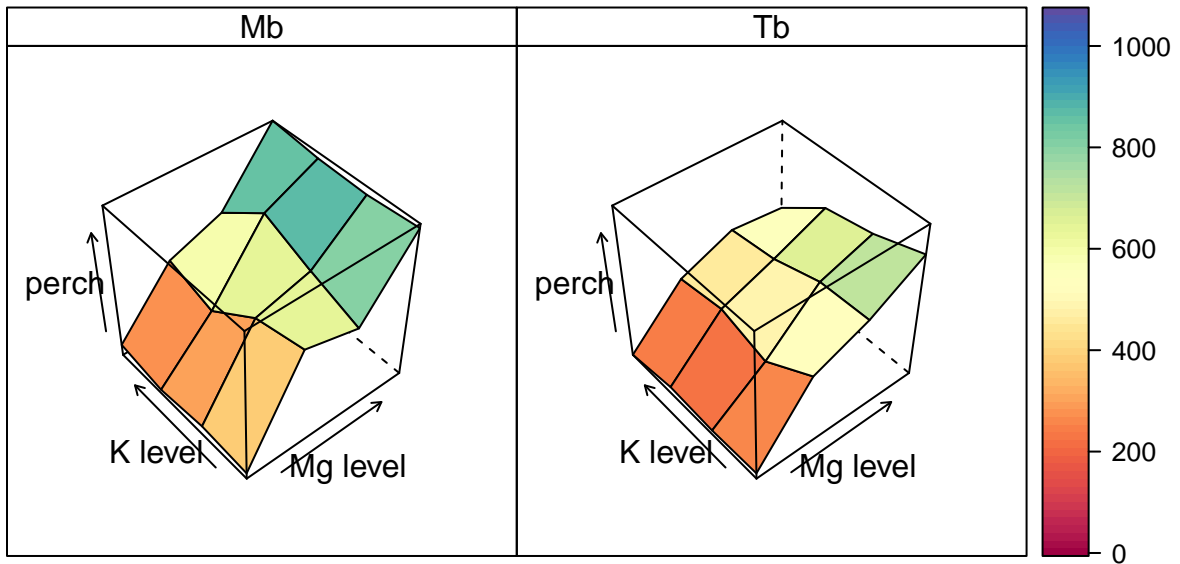

Supplement: Supplementary file 6 — Figure S6. Percentage change (perch) in (a) potassium, and (b) magnesium, concentrations in the upper and lower layers of the pot soil, for the 16 treatments with reference to the common control. Explanations of factor levels and perch are as for Fig. 2. [file ECE3-6-125-s006.pdf]
