# Supplementary material for: Limitation of seedling growth by potassium and magnesium supply for two ectomycorrhizal tree species of a Central African rain forest and its implication for their recruitment
Source: Ecol Evol. 2015 Dec 15;6(1):125–42. doi: 10.1002/ece3.1835 (PMC4716515; doi:10.1002/ece3.1835)
Supplement: Supplementary file 7 — Table S1. Seedling sizes for the two tree species Microberlinia bisulcata and Tetraberlinia bifoliolata grown in a K x Mg factorial fertilizer addition experiment at the Mana Nursery near Korup. [file ECE3-6-125-s007.docx]

Table S1. Seedling sizes for the two tree species *Microberlinia bisulcata* and *Tetraberlinia bifoliolata* grown in a K x Mg factorial fertilizer addition experiment at the Mana Nursery near Korup. Variables: D, stem diameter (mm); H, height (cm); LN, leaf number; LA, leaf area (cm^2^). The statistic (Fisher’s variance ratio, *F*) and its significance are shown for the three main factors only: of the four interaction terms, these were very rarely significant and are not shown apart from their number (*NIntS*). Values in the table are untransformed covariate-adjusted means (averaging across the levels of the other factors). LA was not recorded at H1.

|  |  | *Microberlinia* | | | | *Tetraberlinia* | | | |
| --- | --- | --- | --- | --- | --- | --- | --- | --- | --- |
| Factor | Level | D | H | LN | LA | D | H | LN | LA |
| K | 1 | 9.20^b^ | 56.3^b^ | 28.7^a’^ | 484^c′^ | 11.39^a^ | 90.8^a^ | 36.3^a^ | 1413^a^ |
|  | 2 | 9.58^ab^ | 61.6^ab^ | 30.0^ab’^ | 523^bc′^ | 10.92^ab^ | 87.7^ab^ | 35.8^a^ | 1430^a^ |
|  | 3 | 10.23^a^ | 65.4^a^ | 34.6^a’^ | 650^ab′^ | 10.41^bc^ | 83.6^b^ | 35.0^a^ | 1336^a^ |
|  | 4 | 10.15^a^ | 64.5^a^ | 34.0^a’^ | 673^a′^ | 10.32^c^ | 82.7^b^ | 34.1^a^ | 1382^a^ |
| Mg | 1 | 8.61^b^ | 55.2^b^ | 25.8^b^ | 431^c^ | 10.09^b^ | 81.1^b′^ | 33.4^a^ | 1287^a^ |
|  | 2 | 10.09^a^ | 62.3^a^ | 32.9^ab^ | 563^b^ | 10.93^a^ | 87.3^ab′^ | 35.9^a^ | 1390^a^ |
|  | 3 | 10.38^a^ | 66.7^a^ | 34.9^a^ | 697^a^ | 11.23^a^ | 87.8^ab′^ | 35.9^a^ | 1442^a^ |
|  | 4 | 10.07^a^ | 63.5^a^ | 33.6^a^ | 640^ab^ | 10.80^a^ | 88.6^a′^ | 35.9^a^ | 1441^a^ |
| Harvest | 1 | 7.02^c^ | 48.6^b^ | 24.6^b^ | — | 8.76^c^ | 74.9^c^ | 31.1^b^ | — |
|  | 2 | 10.48^b^ | 68.7^a^ | 37.1^a^ | 659^a^ | 10.97^b^ | 86.2^b^ | 36.6^a^ | 1315^b^ |
|  | 3 | 11.86^a^ | 68.5^a^ | 33.8^a^ | 507^b^ | 12.55^a^ | 97.5^a^ | 38.2^a^ | 1465^a^ |
| *F*-value | K | 2.73* | 3.04* | 1.88^ns^ | 2.09^ns^ | 6.85*** | 2.39^o^ | 0.67^ns^ | 0.32^ns^ |
|  | Mg | 11.13*** | 5.35** | 5.83*** | 7.30*** | 6.25*** | 1.97^ns^ | 1.00^ns^ | 1.00^ns^ |
|  | Harvest | 142.0*** | 41.0*** | 18.9*** | 11.7*** | 131.1*** | 28.4*** | 11.7*** | 4.4* |
|  | *NIntS* | 0 | 1 | 0 | 1 | 0 | 0 | 1 | 0 |
| Means that do not share the same superscripted small letters among levels of the same factor are significantly different (*P* ≤ 0.05). The ′-marks to sets of letters indicate that differences are strictly insufficient since *P*(F) was > 0.05. [Error df: Mb, 137; Tb, 140; except for LA, 90 and 92 resp.] Significance levels, *P*(F): ***, ≤ 0.001; ** ≤ 0.01; * 0.05; ^o^ ≤ 0.10; ns > 0.10. | | | | | | | | | |

|  |
| --- |
