# Supplementary material for: Limitation of seedling growth by potassium and magnesium supply for two ectomycorrhizal tree species of a Central African rain forest and its implication for their recruitment
Source: Ecol Evol. 2015 Dec 15;6(1):125–42. doi: 10.1002/ece3.1835 (PMC4716515; doi:10.1002/ece3.1835)
Supplement: Supplementary file 8 — Table S2. (a) Concentrations of macronutrients (mg/g) in stems at one harvest (H2) of seedlings for the two tree species Microberlinia bisulcata and Tetraberlinia bifoliolata grown in a K x Mg factorial fertilizer addition experiment at the Mana Nursery near Korup. (b) Concentrations of macronutrients (mg/g) in roots at one harvest (H2) of seedlings for the two tree species Microberlinia bisulcata and Tetraberlinia bifoliolata grown in a K x Mg factorial fertilizer addition experiment at the Mana Nursery near Korup. [file ECE3-6-125-s008.docx]

Table S2a: Concentrations of macronutrients (mg/g) in stems at one harvest (H2) of seedlings for the two tree species *Microberlinia bisulcata* and *Tetraberlinia bifoliolata* grown in a K x Mg factorial fertilizer addition experiment at the Mana Nursery near Korup. The values in the table are back-transformed covariate-adjusted means. The statistic (Fisher’s variance ratio, *F*) and its significance is shown for the two main factors only: the interaction term was rarely significant (and only then at *P* < 0.05, although once at P < 0.001 just [stem/Tb/K]), and are not shown.

|  |  |  | *Microberlinia* | | | | | *Tetraberlinia* | | | | |
| --- | --- | --- | --- | --- | --- | --- | --- | --- | --- | --- | --- | --- |
| Part | Factor | Level | Ca | K | Mg | N | P | Ca | K | Mg | N | P |
| Stem | K | 1 | 2.97^a^ | 2.27^b^ | 1.80^a^ | 6.97^a^ | 0.218^b^ | 2.07^a^ | 1.62^c^ | 0.64^a^ | 6.38^a^ | 0.231^b^ |
|  |  | 2 | 2.50^a^ | 3.27^a^ | 2.01^a^ | 6.62^a^ | 0.233^ab^ | 2.30^a^ | 2.37^b^ | 0.60^a^ | 6.54^a^ | 0.274^ab^ |
|  |  | 3 | 2.47^a^ | 3.52^a^ | 1.84^a^ | 6.85^a^ | 0.300^a^ | 2.39^a^ | 2.84^a^ | 0.56^a^ | 6.74^a^ | 0.318^a^ |
|  |  | 4 | 2.69^a^ | 3.82^a^ | 1.75^a^ | 6.65^a^ | 0.279^ab^ | 2.15^a^ | 2.50^ab^ | 0.55^a^ | 6.31^a^ | 0.328^a^ |
|  | Mg | 1 | 3.07^a^ | 3.65^a^ | 0.55^c^ | 6.82^a^ | 0.327^a^ | 2.28^ab′^ | 2.22^a^ | 0.27^b^ | 6.59^a^ | 0.271^a^ |
|  |  | 2 | 2.49^a^ | 2.82^a^ | 2.51^b^ | 6.82^a^ | 0.212^b^ | 1.95^b′^ | 2.25^a^ | 0.72^a^ | 6.28^a^ | 0.285^a^ |
|  |  | 3 | 2.50^a^ | 3.14^a^ | 2.95^a^ | 6.66^a^ | 0.261ab | 2.22^ab′^ | 2.33^a^ | 0.80^a^ | 6.66^a^ | 0.272^a^ |
|  |  | 4 | 2.58^a^ | 3.09^a^ | 2.85^a^ | 6.78^a^ | 0.235^b^ | 2.49^a′^ | 2.33^a^ | 0.78^a^ | 6.45^a^ | 0.313^a^ |
|  | *F*-values | K | 1.31^ns^ | 15.04*** | 1.60ns | 1.07^ns^ | 2.96* | 0.93^ns^ | 27.46*** | 1.71^ns^ | 1.61^ns^ | 3.53* |
|  |  | Mg | 2.13^ns^ | 2.80^ns^ | 73.46*** | 0.17^ns^ | 3.80* | 1.92^ns^ | 0.33^ns^ | 87.70*** | 1.12^ns^ | 0.64^ns^ |
| Means that do not share the same superscripted small letters among levels of the same factor are significantly different (*P* ≤ 0.05). The ′-marks to sets of letters indicate that differences are strictly insufficient since *P*(F) was > 0.05. [Error df: Mb, 41; Tb, 42.] Significance levels, *P*(F): ***, ≤ 0.001; ** ≤ 0.01; * 0.05; ^o^ ≤ 0.10; ns > 0.10. | | | | | | | | | | | | |

Table S2b: Concentrations of macronutrients (mg/g) in roots at one harvest (H2) of seedlings for the two tree species *Microberlinia bisulcata* and *Tetraberlinia bifoliolata* grown in a K x Mg factorial fertilizer addition experiment at the Mana Nursery near Korup. The values in the table are back-transformed covariate-adjusted means. The statistic (Fisher’s variance ratio, *F*) and its significance is shown for the two main factors only: the interaction term was rarely significant (and only then at *P* < 0.05), and are not shown.

|  |  |  | *Microberlinia* | | | | | *Tetraberlinia* | | | | |
| --- | --- | --- | --- | --- | --- | --- | --- | --- | --- | --- | --- | --- |
| Part | Factor | Level | Ca | K | Mg | N | P | Ca | K | Mg | N | P |
| Roots | K | 1 | 2.45^a′^ | 2.59^b^ | 1.99^ab^ | 7.84^a^ | 0.365^a^ | 1.16^b^ | 1.99^b^ | 0.76a | 8.85^b′^ | 0.391^b^ |
|  |  | 2 | 1.91^b′^ | 3.32^a^ | 2.13^a^ | 8.36^a^ | 0.426^a^ | 1.29^ab^ | 2.58a | 0.81a | 9.86^a′^ | 0.447^a^ |
|  |  | 3 | 2.02^ab′^ | 3.29^a^ | 1.95^ab^ | 8.01^a^ | 0.427^a^ | 1.46^a^ | 2.78^a^ | 0.77^a^ | 9.61^ab′^ | 0.448^a^ |
|  |  | 4 | 2.32^ab′^ | 3.56^a^ | 1.79^b^ | 7.84^a^ | 0.399^a^ | 1.10^b^ | 2.72^a^ | 0.75^a^ | 9.36^ab′^ | 0.462^a^ |
|  | Mg | 1 | 2.86^a^ | 3.43^a′^ | 0.82^c^ | 8.69^a^ | 0.462^a^ | 1.50^a^ | 2.50ab′ | 0.45^c^ | 9.51^a^ | 0.445^a^ |
|  |  | 2 | 2.14^b^ | 3.07^ab′^ | 2.36^b^ | 7.71^b^ | 0.373^b^ | 1.13^b^ | 2.50^ab′^ | 0.82^b^ | 9.27^a^ | 0.417^a^ |
|  |  | 3 | 1.91^b^ | 3.00^b′^ | 2.55^ab^ | 7.68^b^ | 0.381^b^ | 1.10^b^ | 2.37^b′^ | 0.84^a^ | 9.12^a^ | 0.426^a^ |
|  |  | 4 | 1.88^b^ | 3.20^ab′^ | 3.00^a^ | 8.02^ab^ | 0.405^ab^ | 1.29^ab^ | 2.62a′ | 0.98^a^ | 9.74^a^ | 0.457^a^ |
|  | *F*-values | K | 1.13^ns^ | 14.27*** | 3.11* | 1.27^ns^ | 1.81^ns^ | 4.00* | 26.42*** | 2.19^ns^ | 1.92^ns^ | 4.11* |
|  |  | Mg | 4.69** | 2.16^ns^ | 96.81*** | 3.99* | 3.36* | 5.33** | 1.92^ns^ | 162.59*** | 0.72^ns^ | 1.25^ns^ |
| Means that do not share the same superscripted small letters among levels of the same factor are significantly different (*P* ≤ 0.05). The ′-marks to sets of letters indicate that differences are strictly insufficient since *P*(F) was > 0.05. [Error df: Mb, 40; Tb, 39.] Significance levels, *P*(F): ***, ≤ 0.001; ** ≤ 0.01; * 0.05; ^o^ ≤ 0.10; ns > 0.10. | | | | | | | | | | | | |
