# Supplementary material for: Limitation of seedling growth by potassium and magnesium supply for two ectomycorrhizal tree species of a Central African rain forest and its implication for their recruitment
Source: Ecol Evol. 2015 Dec 15;6(1):125–42. doi: 10.1002/ece3.1835 (PMC4716515; doi:10.1002/ece3.1835)
Supplement: Supplementary file 9 — Table S3. (a)Concentrations of three cations (μg/g) in the upper soil layer of pots with seedlings of Microberlinia bisulcata and Tetraberlinia bifoliolata grown in the K x Mg factorial fertilizer addition experiment. (b) Concentrations of three cations (μg/g) in the lower soil layer of pots with seedlings of Microberlinia bisulcata and Tetraberlinia bifoliolata grown in the K x Mg factorial fertilizer addition experiment. [file ECE3-6-125-s009.docx]

Table S3a. Concentrations of three cations (μg/g) in the upper soil layer of pots with seedlings of *Microberlinia bisulcata* and *Tetraberlinia bifoliolata* grown in the K x Mg factorial fertilizer addition experiment. The values in the table are back-transformed means. The statistic (Fisher’s variance ratio, *F*) and its significance is shown for the three main factors only: of the four interaction terms, these were not often significant (and only then at *P* < 0.01) and are not shown apart from their number (*NIntS*).

|  |  | *Microberlinia* | | | *Tetraberlinia* | | |
| --- | --- | --- | --- | --- | --- | --- | --- |
| Factor | Level | Ca | K | Mg | Ca | K | Mg |
| K | 1 | 35.41^a^ | 19.24^c^ | 20.55^b^ | 26.44^b′^ | 20.15^c^ | 28.73^b^ |
|  | 2 | 34.67^a^ | 24.98^b^ | 26.90^a^ | 28.82^ab′^ | 30.60^b^ | 28.02^b^ |
|  | 3 | 37.64^a^ | 30.72^b^ | 28.39^a^ | 31.37^ab′^ | 33.65^b^ | 37.52^a^ |
|  | 4 | 38.90^a^ | 38.74^a^ | 32.04^a^ | 33.08^a′^ | 41.26^a^ | 34.95^ab^ |
| Mg | 1 | 29.17^b^ | 34.92^a^ | 7.46^d^ | 24.80^b^ | 35.98^a^ | 8.51^c^ |
|  | 2 | 35.45^b^ | 25.10^b^ | 26.52^c^ | 27.66^ab^ | 29.90^b^ | 36.02^b^ |
|  | 3 | 37.30^ab^ | 26.39^b^ | 44.21^b^ | 31.53^a^ | 26.00^b^ | 55.70^a^ |
|  | 4 | 46.57^a^ | 24.73^b^ | 57.57^a^ | 36.31^a^ | 30.00^ab^ | 59.15^a^ |
| Harvest | 1 | 18.21^b^ | 18.69^b^ | 21.28^b^ | 14.75^b^ | 24.58^b^ | 27.72^b^ |
|  | 2 | 73.55^a^ | 40.49^a^ | 33.34^a^ | 66.81^a^ | 38.17^a^ | 37.68^a^ |
| *F*-values | K | 0.37^ns^ | 16.04*** | 8.68*** | 1.81^ns^ | 24.37*** | 3.69* |
|  | Mg | 4.82** | 4.72** | 203.24*** | 4.37** | 4.69** | 119.41*** |
|  | Harvest | 253.3*** | 108.0*** | 49.9*** | 454.5*** | 53.2*** | 10.9*** |
|  | *NIntS* | 1 | 1 | 0 | 2 | 2 | 0 |
| Means that do not share the same superscripted small letters among levels of the same factor are significantly different (P ≤ 0.05). The ′-marks to sets of letters indicate that differences are strictly insufficient since *P*(F) was > 0.05. [Error df: Mb, 88; Tb, 85.] Significance levels, *P*(F): ***, ≤ 0.001; ** ≤ 0.01; * 0.05; ^o^ ≤ 0.10; ns > 0.10. | | | | | | | |

Table S3b. Concentrations of three cations (μg/g) in the lower soil layer of pots with seedlings of *Microberlinia bisulcata* and *Tetraberlinia bifoliolata* grown in the K x Mg factorial fertilizer addition experiment. The values in the table are back-transformed means. The statistic (Fisher’s variance ratio, *F*) and its significance is shown for the three main factors only: of the four interaction terms, these were not often significant (and only then at *P* < 0.01) and are not shown apart from their number (*NIntS*).

|  |  | *Microberlinia* | | | *Tetraberlinia* | | |
| --- | --- | --- | --- | --- | --- | --- | --- |
| Factor | Level | Ca | K | Mg | Ca | K | Mg |
| K | 1 | 35.48^a^ | 21.49^d^ | 32.33^a^ | 44.84^a^ | 16.79c | 56.37^a^ |
|  | 2 | 31.44^a^ | 43.29^c^ | 34.23^a^ | 42.22^a^ | 46.43^b^ | 47.99^a^ |
|  | 3 | 34.99^a^ | 63.69^b^ | 29.76^a^ | 39.33^a^ | 67.09^ab^ | 47.56^ab^ |
|  | 4 | 35.59^a^ | 88.41^a^ | 32.72^a^ | 37.52^a^ | 80.64^a^ | 37.41^b^ |
| Mg | 1 | 36.63^a^ | 58.56^a^ | 7.83^c^ | 46.34^a^ | 56.94^a^ | 12.00^c^ |
|  | 2 | 34.50^a^ | 47.42^ab^ | 39.13^b^ | 44.70^a^ | 48.38^ab^ | 55.42^b^ |
|  | 3 | 32.72^a^ | 50.00^a^ | 49.21^b^ | 36.42^b^ | 39.85^bc^ | 79.76^a^ |
|  | 4 | 33.58^a^ | 37.75^b^ | 71.38^a^ | 36.97^ab^ | 38.51^c^ | 91.10^a^ |
| Harvest | 1 | 18.73^b^ | 39.49^b^ | 33.28^a^ | 27.63^b^ | 42.61^b^ | 48.91^a^ |
|  | 2 | 62.93^a^ | 57.97^a^ | 31.19^a^ | 67.63^a^ | 49.16^a^ | 44.75^a^ |
| *F*-values | K | 0.50^ns^ | 37.07*** | 0.41^ns^ | 1.10^ns^ | 101.96*** | 3.95* |
|  | Mg | 0.34^ns^ | 3.30* | 113.26*** | 3.15* | 6.07*** | 114.95*** |
|  | Harvest | 211.6*** | 14.8*** | 0.49^ns^ | 149.4*** | 2.99^o^ | 1.56^ns^ |
|  | *NIntS* | 0 | 0 | 0 | 0 | 3 | 0 |
| Means that do not share the same superscripted small letters among levels of the same factor are significantly different (*P* ≤ 0.05). [Error df: Mb, 88; Tb, 82.] Significance levels, *P*(F): ***, ≤ 0.001; ** ≤ 0.01; * 0.05; ^o^ ≤ 0.10; ns > 0.10. | | | | | | | |
