# Supplementary material for: Limitation of seedling growth by potassium and magnesium supply for two ectomycorrhizal tree species of a Central African rain forest and its implication for their recruitment
Source: Ecol Evol. 2015 Dec 15;6(1):125–42. doi: 10.1002/ece3.1835 (PMC4716515; doi:10.1002/ece3.1835)
Supplement: Supplementary file 10 [file ECE3-6-125-s010.pdf]

Legends to SM Figures

Figure S1. *Microberlinia bisulcata* (Mb) and *Tetraberlinia bifoliolata* (Tb) growing within the shade house at the Mana Bridge Nursery, next to Korup, Ndian.

Figure S2. Percentage change (*perch*) in dry mass of the two species *Microberlinia bisulcata* (Mb) and *Tetraberlinia bifoliolata* (Tb) for the 16 treatments (four levels of K x four levels for Mg; each factor being null and three increasing additions), averaged across the three harvests, with reference to the common control [1,1] for (a) root, and (b) shoot parts. This complements leaf and total parts in Fig. 2.

Figure S3. Mean concentrations (mg/g) of potassium and magnesium, in leaves (green), roots (brown) and stems (light blue bars) of seedlings of *Microberlinia bisulcata* (Mb) and *Tetraberlinia bifoliolata* (Tb) at harvest 2 for the secondary treatments. A secondary treatment had no factor at level 1. Concentrations for corresponding primary treatments are shown in Fig. 3.

Figure S4. Mean concentrations (mg/g) of (a) calcium, (b) nitrogen, and (c) phosphorus, in leaves (green), roots (brown) and stems (light blue bars) of seedlings of *Microberlinia bisulcata* (Mb) and *Tetraberlinia bifoliolata* (Tb) at harvest 2 for the primary and secondary treatments. A primary treatment had at least one factor at level 1, whilst secondary treatments had none.

Figure S5. Percentage change (*perch*) in calcium, nitrogen and phosphorus leaf nutrient concentrations of *Microberlinia bisulcata* (Mb) and *Tetraberlinia bifoliolata* (Tb) for the 16 treatments with reference to the common control. Explanations of factor levels and *perch* are given in Fig. 2.

Figure S6. Percentage change (*perch*) in (a) potassium, and (b) magnesium, concentrations in the upper and lower layers of the pot soil, for the 16 treatments with reference to the common control. Explanations of factor levels and *perch* are as for Fig. 2.
